# Insights into molecular mechanisms of drug metabolism dysfunction of human CYP2C9*30

**DOI:** 10.1371/journal.pone.0197249

**Published:** 2018-05-10

**Authors:** Maxime Louet, Céline M. Labbé, Charline Fagnen, Cassiano M. Aono, Paula Homem-de-Mello, Bruno O. Villoutreix, Maria A. Miteva

**Affiliations:** 1 Université Paris Diderot, Sorbonne Paris Cité, Inserm UMR-S 973, Molécules Thérapeutiques *In silico*, Paris, France; 2 INSERM, U973, Paris, France; 3 Université Pierre et Marie Curie, Sorbonne Universités, UMR 7590, Institut de Minéralogie, de Physique des Matériaux et de Cosmochimie, Paris, France; 4 Universidade Federal do ABC, Centro de Ciências Naturais e Humanas, Santo André, Brazil; Hong Kong University of Science and Technology, HONG KONG

## Abstract

Cytochrome P450 2C9 (CYP2C9) metabolizes about 15% of clinically administrated drugs. The allelic variant CYP2C9*30 (A477T) is associated to diminished response to the antihypertensive effects of the prodrug losartan and affected metabolism of other drugs. Here, we investigated molecular mechanisms involved in the functional consequences of this amino-acid substitution. Molecular dynamics (MD) simulations performed for the active species of the enzyme (heme in the Compound I state), in the apo or substrate-bound state, and binding energy analyses gave insights into altered protein structure and dynamics involved in the defective drug metabolism of human CYP2C9.30. Our data revealed an increased rigidity of the key Substrate Recognition Sites SRS1 and SRS5 and shifting of the β turn 4 of SRS6 toward the helix F in CYP2C9.30. Channel and binding substrate dynamics analyses showed altered substrate channel access and active site accommodation. These conformational and dynamic changes are believed to be involved in the governing mechanism of the reduced catalytic activity. An ensemble of representative conformations of the WT and A477T mutant properly accommodating drug substrates were identified, those structures can be used for prediction of new CYP2C9 and CYP2C9.30 substrates and drug-drug interactions.

## Introduction

Cytochrome P450 2C9 (CYP2C9) is the most expressed member of the human CYP2C family and metabolizes more than 15% of clinically administrated drugs including hypoglycemic agents, anticonvulsants, anticoagulants, nonsteroidal anti-inflammatory drugs (NSAIDs), antihypertensives, diuretic drugs [1–3] and several endogenous compounds [4]. The genetic variations resulting mainly from single nucleotide polymorphisms (SNPs) explain a large part of the inter-individual variability in the activity of CYP enzymes [5]. More than 50 variants of CYP2C9 have shown decreased enzymatic activity [3, 6–8]. Polymorphisms of *CYP2C9* can affect the clinical response of drugs metabolized by CYP2C9, especially those with a narrow therapeutic index and can induce adverse drug reactions (ADR). The rare allelic variant CYP2C9*30 found in Japanese subjects has been associated to diminished response to the antihypertensive effects of the prodrug losartan [9]. Moreover, in vitro experiments have shown that the intrinsic clearance of losartan, diclofenac and glimepiride was markedly decreased for CYP2C9.30 (mutation A477T) (by > 70%) compared to that of the wild-type (WT) enzyme [10, 11]. It has been previously proposed that conformational changes occurring nearby F476 could play a role in the substrate recognition [12]. Recently the crystal structures of human CYP2C9 and its variant CYP2C9.30 co-crystalized with losartan have been reported [13]. However, the structural overlay of these two complexes did not reveal significant differences between the losartan-bound WT and A477T mutant crystal structural (Root Mean Square Deviation (RMSD) of ~0.2 Å). Three losartan molecules have been found to bind in the same positions in the co-crystallized WT and A477 mutant structures, neither of them was oriented with the losartan hydroxylation site toward the heme iron required for the hydroxylation reaction. Thus, the molecular mechanisms responsible for the mutation-induced dysfunction of the metabolism of CYP2C9.30 remain unclear.

**Fig 1:**
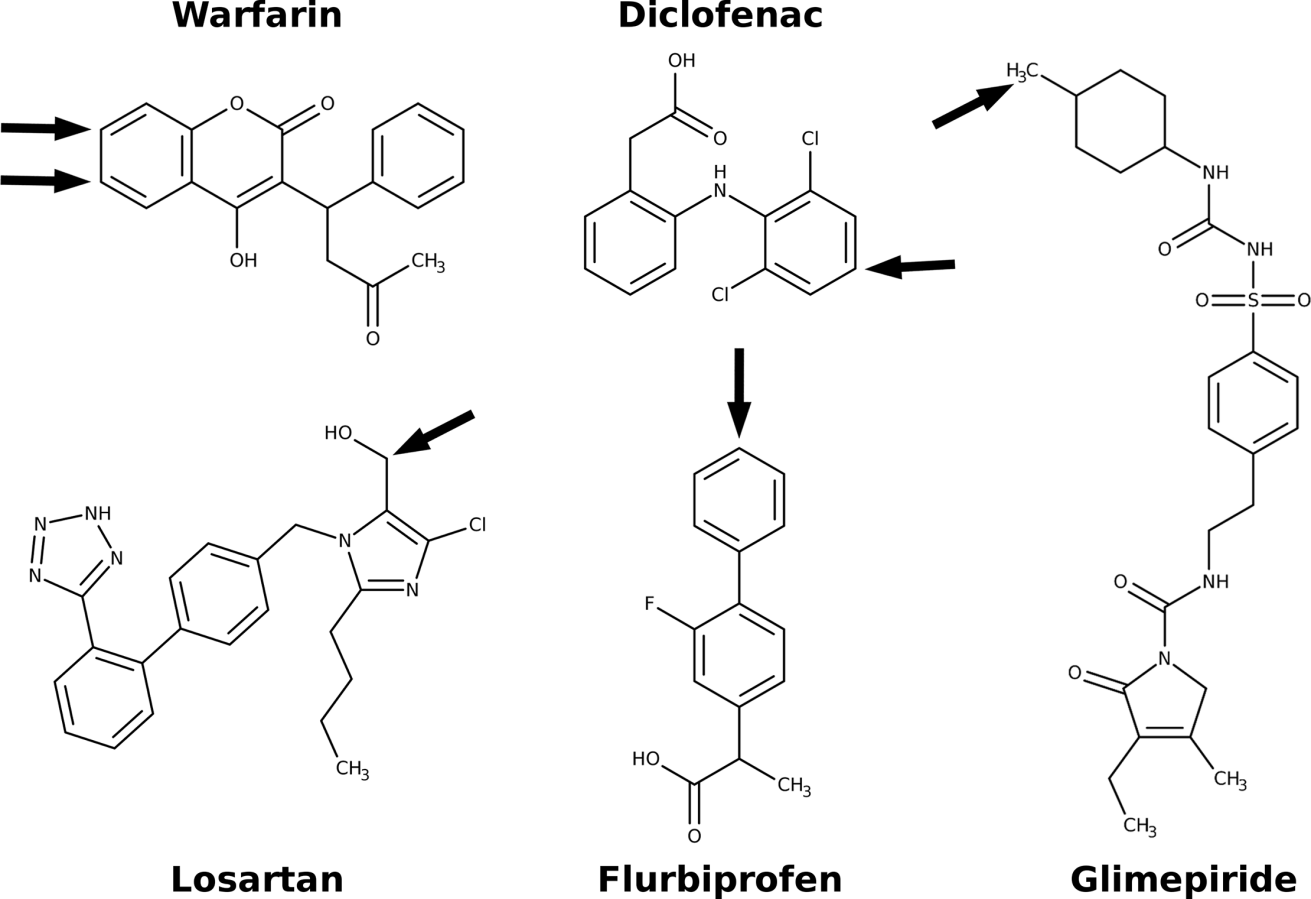
Structures of five drug substrates of CYP2C9. The arrows show the sites of metabolism mediated by CYP2C9.

Here we provide insights into the dysfunctional metabolism mechanism in human CYP2C9.30 based on molecular dynamics (MD), quantum mechanics and docking simulations. Molecular dynamics (MD) simulations combined with energetic analyses have been demonstrated to be a pertinent approach to understand molecular mechanisms of amino-acid substitutions affecting drug metabolism or regioselectivity of CYP [14–18]. In this work, we explored a large region of the conformational space of the WT of CYP2C9 and its mutant A477T, in the apo or substrate-bound states, for two chemically different substrates, diclofenac and losartan, with the site of hydroxylation oriented toward the heme iron consistent with the metabolic reaction. MD simulations of 1.5 μs were run in total. Structural and dynamics changes caused by the A447T mutation were revealed and analyzed to explain the decrease of the enzymatic activity of CYP2C9.30. The A477T mutant demonstrated an increased rigidity of key segments involved in substrate recognition that could affect the substrate entrance and accommodation. Binding energy analyses via molecular docking of five drugs substrates of CYP2C9 (shown in Fig 1) suggested molecular mechanisms implicated in the defective drug metabolism in carriers of CYP2C9*30. Our results highlighted conformational and dynamic changes believed to be involved in the governing mechanism of the catalytic activity reduction and confirmed that protein dynamics affected by missense mutations could play a crucial role in affected regulation of substrate entry, recognition and metabolism of CYP2C9.

## Results and discussion

### Conformational and dynamics behavior of CYP2C9 WT and A477T

The A477T mutation present in CYP2C9.30 variant is located in the Substrate Recognition Site 6 (SRS6) (Supplementary S1 Fig). The comparison of the two crystal structures of losartan-bound CYP2C9 WT and A477T mutant did not show important differences between the two complexes except a rotation of the side chain of Q214 forming thus a hydrogen bond (HB) with the site chain of T477 in the mutant protein (PDB IDs 5XXI and 5X23 [13]). Three losartan molecules were found to bind equally in the crystal structures of CYP2C9 WT and A477T mutant, two substrate molecules were present in the binding pocket and one of them was located in a peripheral site of the protein. One of the co-crystalized losartan molecule was bound far from the heme but nearby Q214 and the mutation residue 477 and involved in tight aromatic contacts with F476. The presence of the losartan molecule at this place does not facilitate understanding the role of the mutated residue 477 and its interactions with Q214 and F476. The residue 477 shows a low sequence conservation based on a sequence alignment of all CYP2 family members performed with the ConSurf web server [19]. A recent analysis of this mutation [3] carried out with well-established online computational tools predicting mutation effects [20–23] did not suggested a hypothesis about its impact on the enzyme structure or function. To elucidate the molecular mechanisms involved in the defected metabolism of CYP2C9.30 we performed molecular dynamics simulations of the active species of the WT of CYP2C9 and the mutant A477T, both containing the heme in the Compound I state (Cpd I) [24]. To better understand the structural and dynamics bases for the metabolism alteration we have studied six CYP2C9 systems (1) WT apo, (2) A477T apo, (3) WT diclofenac-bound, (4) A477T diclofenac-bound, (5) WT losartan-bound and (6) A477T losartan-bound. The initial positions of the bound substrates were obtained by docking and corresponded to the Sites of Metabolism (SOM) oriented toward the oxygen atom of Cpd I. It is to note that the positions of the co-crystalized losartan in the WT and A477T mutant structures are not consistent with the catalytic reaction because the site of hydroxylation is positioned away from the heme iron [13]. Thus, these crystal binding modes are not appropriate for our MD simulations. For each of the six systems, 5 MD trajectories of 50 ns were run. The MD simulations of 250 ns per studied system have shown stable protein structures of the CYP2C9 WT and its A477T mutant. The all-heavy-atom RMSD over the merged five MD trajectories for each system showed maximal mean value of 1.59 Å for all systems. The backbone RMSD data (see S2 Fig) did not show large conformational changes of the A477 mutant compared to the WT suggesting that subtle molecular mechanisms could be responsible for the altered metabolism.

**Fig 2:**
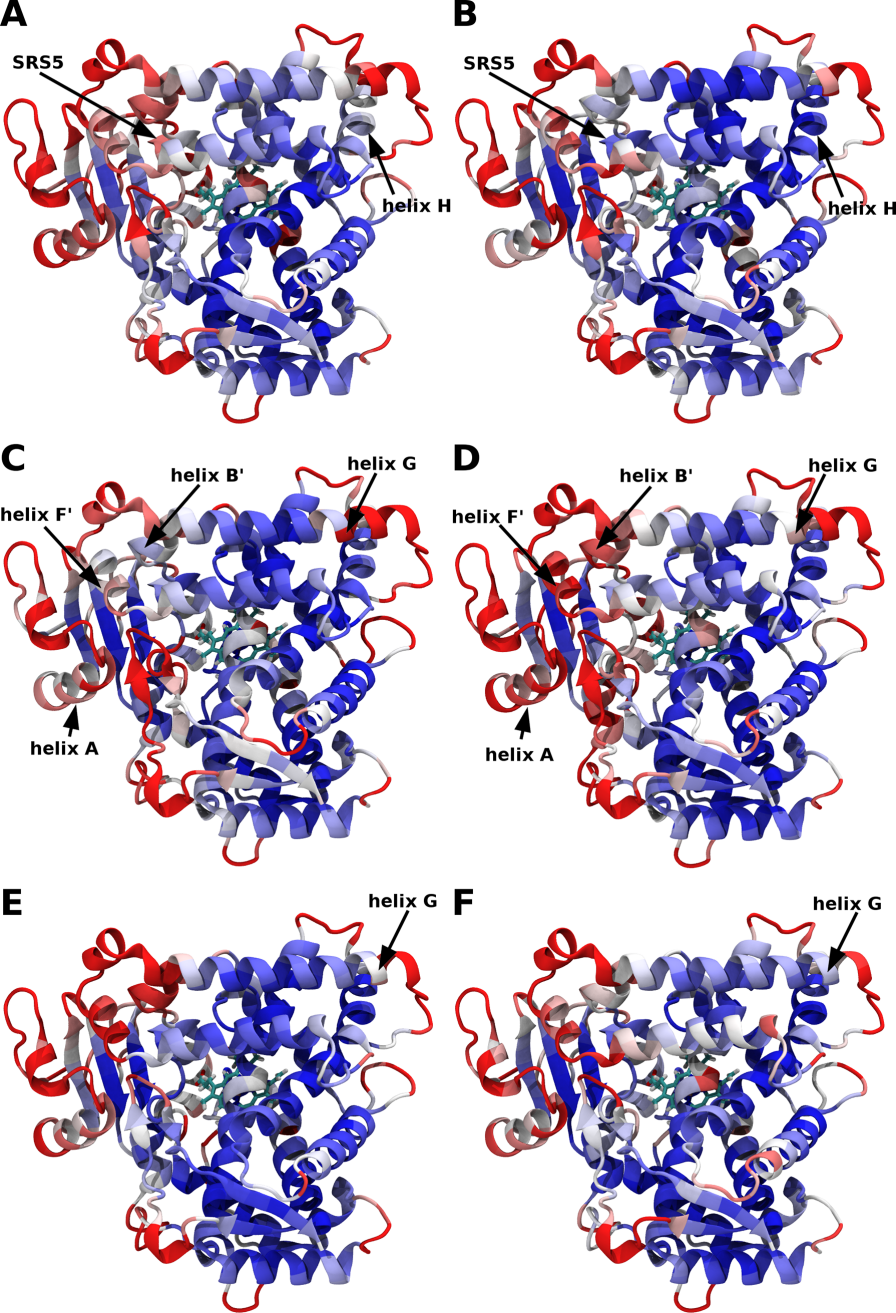
Root Mean Square Fluctuations (RMSF) of the Cα atoms during the MD simulations of CYP2C9. The RMSF computed for five merged MD trajectories of (A) WT apo, (B) A477T apo, (C) WT diclofenac-bound, (D) A477T diclofenac-bound, (E) WT losartan-bound and (F) A477T losartan-bound are mapped on the CYP2C9 crystallographic structure (PDB ID: 1OG5) [25]. The color code of RMSF is ranging from 0.7 Å (blue) to 1.6 Å (red). The black arrows show noticeable differences between the WT and the mutant RMSF.

The Root Mean Square Fluctuations (RMSF) results confirmed the stability of the WT and mutant A477T structures during the MD simulations. The mean RMSF values of the merged MD trajectories for each of the six systems varied between 1.08 Å and 1.18 Å. However, the comparison of the atomic fluctuations per residue (see Fig 2 and S3 Fig) revealed several discrepancies between the WT apo and the mutant apo proteins in key regions involved in substrate binding. The A447T mutant in apo form was found to be more rigid than the WT apo CYP2C9, in particular the residues from 367 to 369 of SRS5, and the residues from 271 to 276 of helix H (see Fig 2A and 2B). It is important to note that in the presence of a bound substrate (see Fig 2C, 2D, 2E and 2F), the WT and the mutant proteins showed similar flexibility probably due to the stabilization of the protein structures triggered by the interactions with the ligand. Different regions were influenced when CYP2C9 was bound to diclofenac or losartan that was likely due to the different locations of the two substrates in the binding pocket during the MD simulations (see below on the substrates dynamics).

**Fig 3:**
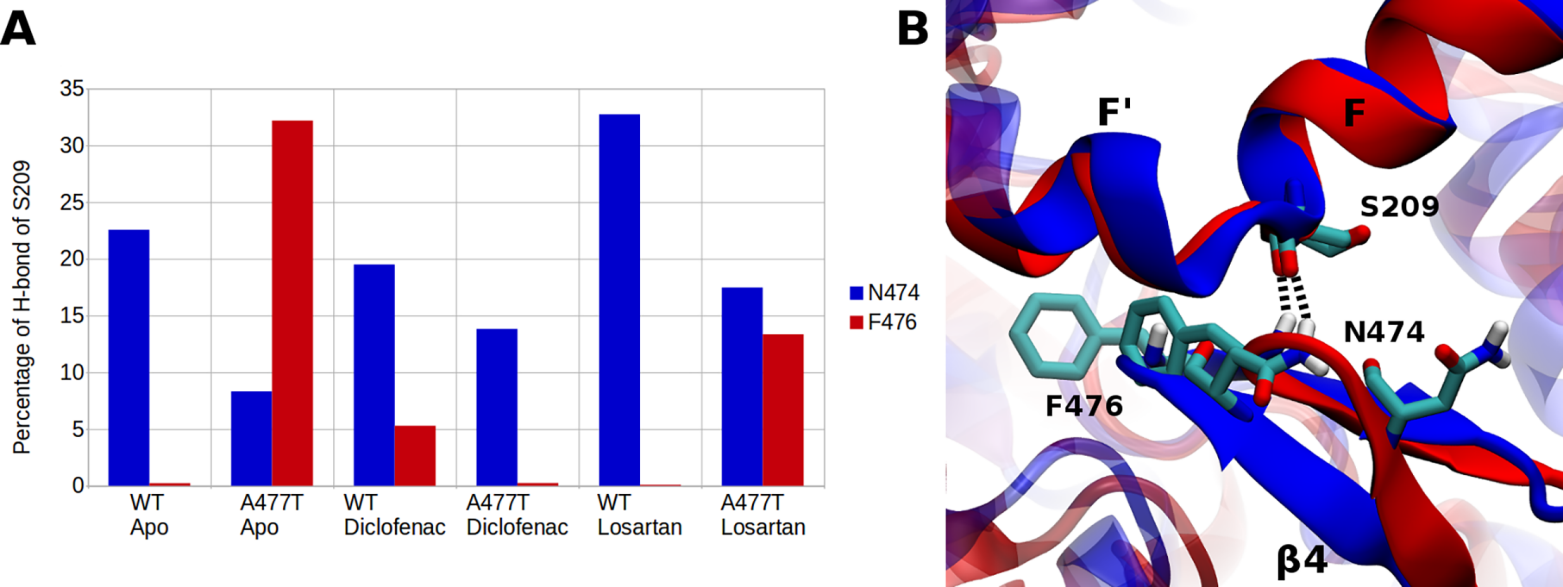
Interaction patterns between the helix F and the β-turn 4. (A) Percentage of the HBs between S209 and N474 or F476 during the MD simulations. (B) Representation of the shift motion of the β-turn 4 in regard to helix F. The interactions of S209 with N474 (blue) and F476 (red) are noted.

In order to find noticeable conformational differences of the substrate binding pocket of CYP2C9 between the WT and the A477T mutant, we performed structural clustering of the five merged MD trajectories for each of the studied systems based on the binding site residues (see S4 Fig). The structural analysis of the centroid structures for the 15 most populated clusters of the six studied systems covering at least 55% of the binding site conformational space explored during all MD simulations showed structural differences of the β turn 4 including the mutation site. For the WT apo state, the most representative centroid structure corresponding to 11.3% of the total 250 ns MD was very similar to the initial apo crystal structure of CYP2C9 PDB ID 1OG2 [25]), (RMSD of the Cα atoms of 1.01 Å) with a conserved β turn 4 conformation and unchanged F100 and F114 side-chain orientations. The other centroids showed different side-chain orientations for F100 and F114 belonging to the BC loop, a protein segment known to be a part of previously identified substrate access channels (2a, 2b, 2c, 2ac, and 2e) [26, 27] (see below for channel dynamics). The 15 centroid structures of the A477T apo mutant showed different binding-site conformations for F476 belonging to the ß turn 4. F476 has already been pointed out to be a key residue involved in the channel access to the catalytic pocket [25, 27]. To study the conformational changes of the ß turn 4 we further analyzed the HB interactions of the β turn 4 during all MD simulations. The most important changes were detected for the residue S209 located on helix F with the residues 470–490 of the β turn 4. Two residues were identified to be involved in HBs with S209 (Fig 3A). We did not observe a HB bond between Q214 and T477 during the mutant apo MD simulations. In the apo mutant protein we detected possible HB breaks between the side chain of Q214 and the main chains of G475 (a HB conserved in 10.3% in the apo WT *vs* 5.6% in the apo mutant MD simulations) and F476 (a HB conserved in 26.3% in the apo WT *vs* 2.1% in the apo mutant MD simulations). Changes of the HB interactions of Q214 due to the A477 mutation were also observed in the case of bound losartan to CYP2C9 [13]. Therefore, significant interaction modifications occur in the mutant protein compared to the WT one corresponding to a movement of the β turn 4 in regard to the helix F of about 5 Å (see Fig 3B). This shift may alter the well-known channel access including F476 (or so-called warfarin-binding subpocket found in the co-crystalized CYP2C9 –warfarin structure, PDB ID 1OG5 [25]) and may destabilize the CYP2C9-substrate interactions in this zone.

**Fig 4:**
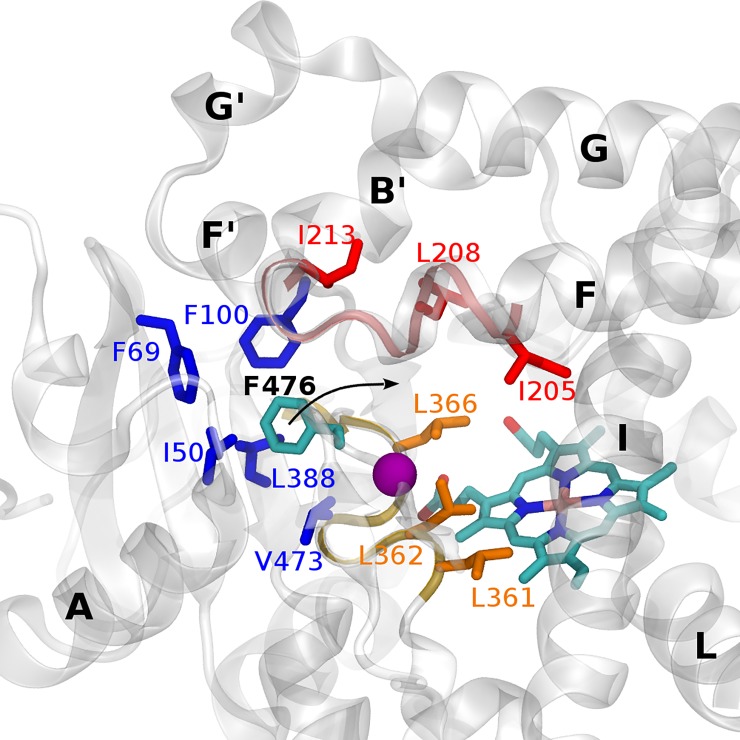
Hydrophobic contacts of F476 and other residues of the CYP2C9 binding pocket monitored over the MD simulations. Residues found in contact with F476 are depicted in sticks. Three distinct hydrophobic clusters are found and colored in blue, red and orange. The purple sphere represents the location of the mutation.

Hydrophobic interactions between F476 and other hydrophobic residues of the CYP2C9 binding pocket were monitored within a distance of 5 Å during the MD simulations (see Fig 4 and S5 Fig). We observed that in the presence of the A477T mutation, F476 increased its contacts with L361 and F476 remained in stable contacts with L366 in the cases of ligand-bound states. Overall, F476 showed more stable contacts with L361, L362 and L366 belonging to SRS5 (shown in orange in Fig 4) in the mutant protein than in the WT one. These contacts explain the increased rigidity of SRS5 in the mutant compared to the WT (S3 Fig). When focusing on the apo state, we observed that the shift motion of the β turn 4 in the mutant protein modified the hydrophobic-interaction patterns of F476 as well. It is important to note that new contacts of F476 and L208 and L362 appeared during the MD simulations, which were not seen in the X-ray structure. The contacts of F476 with L208 strongly increased in the mutant structures confirming the movement of F476 toward L208 (helix F shown in red in Fig 4). This is likely related to the partial closing of the solvent channel S1 in the presence of the mutation A477T (see details on the channels dynamics below). Interestingly, F476 remained in stable contacts with the key F100 gate residue in the apo WT and mutant protein. This interaction is also conserved in the two X-ray structures (warfarin-bound PDB ID 1OG5 and flurbiprofen-bound PDB ID 1R9O).

### Substrate dynamics during the MD simulations

**Fig 5:**
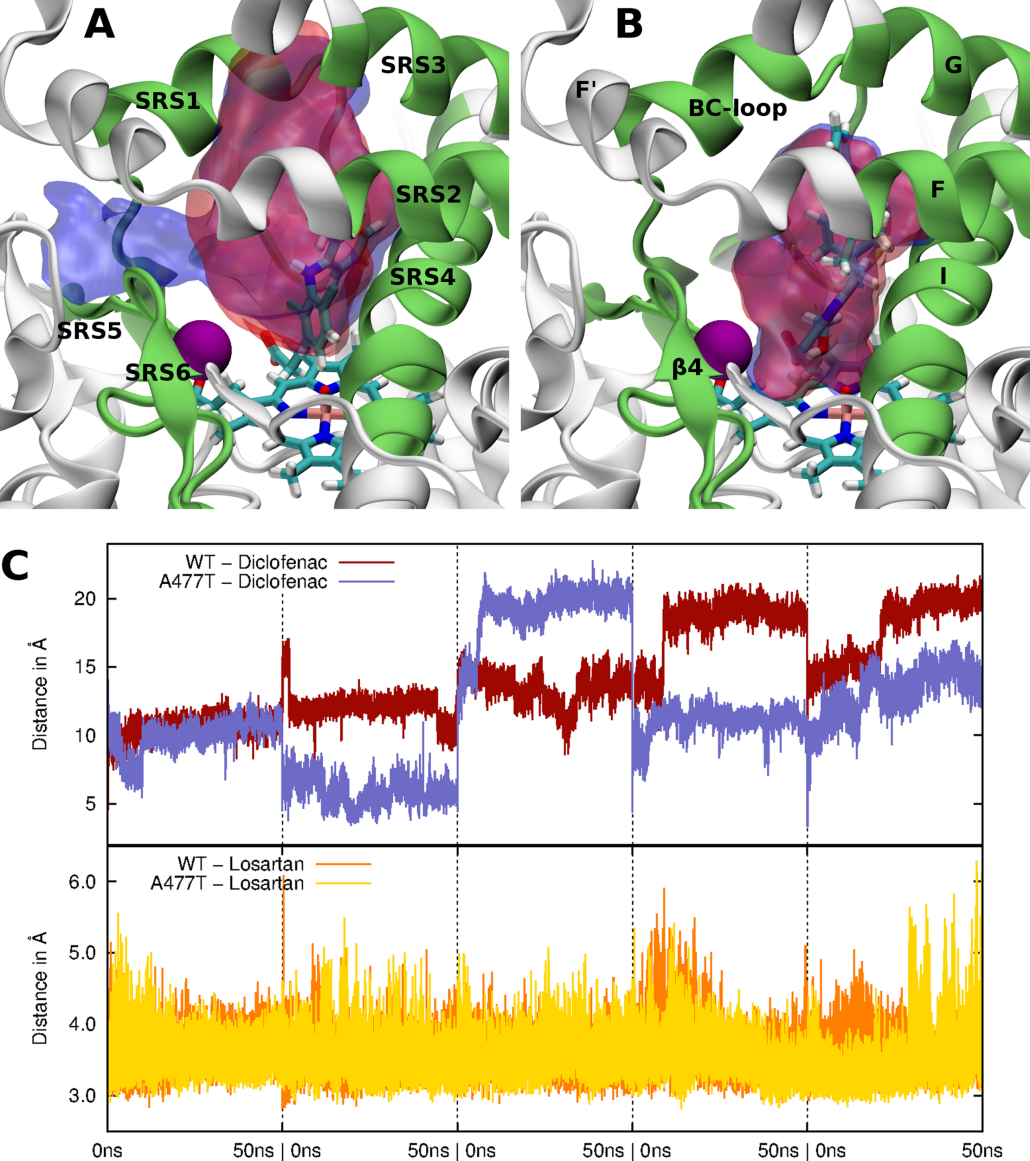
Substrate dynamics during the MD simulations for the WT and the A477T mutant. Volumetric map of the pocket space explored by diclofenac (A) and losartan (B) during the entire MD simulations are shown. The initial positions of the ligands and Cpd I are shown as sticks. The protein is represented as a white cartoon. SRS regions are colored in green. The purple sphere represents the location of the mutation. The blue (WT) and red (A477T) surfaces represent regions explored by the ligands over 95% of all simulation snapshots. (C) Distance between the Site Of Metabolism (SOM) of diclofenac (top) and losartan (bottom) and the reactive oxygen atom of Cpd I along the MD simulations.

Substrate positions are crucial for the oxidation reaction. Fig 5 show the diclofenac and losartan dynamics during the MD simulations for the WT and the A477T mutant. The diclofenac moved quickly out of its initial position and explored the entire binding pocket during three out of the five simulations for both WT and its A477T mutant (see Fig 5C). Despite of its mobility, the diclofenac always remained in contact with one of the SRS1, SRS2, SRS3, and SRS4 regions during both WT and A477T mutant simulations. However, the diclofenac lost completely its contacts with SRS5 during the mutant MD simulations (see Fig 5A).

On the contrary, the losartan reached an equilibrium position (called here the preferential conformation), remaining in the same subpocket in contact with the heme similarly to its initial docking position in both WT and A477T mutant proteins (see Fig 5C and S6 Fig). Along the 250 ns MD simulations for the WT, 1.5%, 86% and 11.5% of all generated conformations corresponded to the initial structure, the preferential structure and other diverse structures, respectively. For the A477T mutant, 8.5%, 52.7% and 38.8% of all MD conformations corresponded to the initial structure, the preferential structure and other structures, respectively. These observations suggested that the preferential losartan position in the catalytic site consistent with the catalytic reaction is more stable in the WT than in the A477T mutant.

The RMSD of Cpd I along the MD simulations (S7 Fig) showed that Cpd I remained slightly less stable in the A477T mutant than in the WT. The Cpd I instability could also alter the catalytic activity of the A477T mutant. Not surprisingly, the interactions between Cpd I and the bound substrates stabilized Cpd I in the holo states in comparison with the apo states.

### Channels dynamics of CYP2C9 WT and A477T

**Fig 6:**
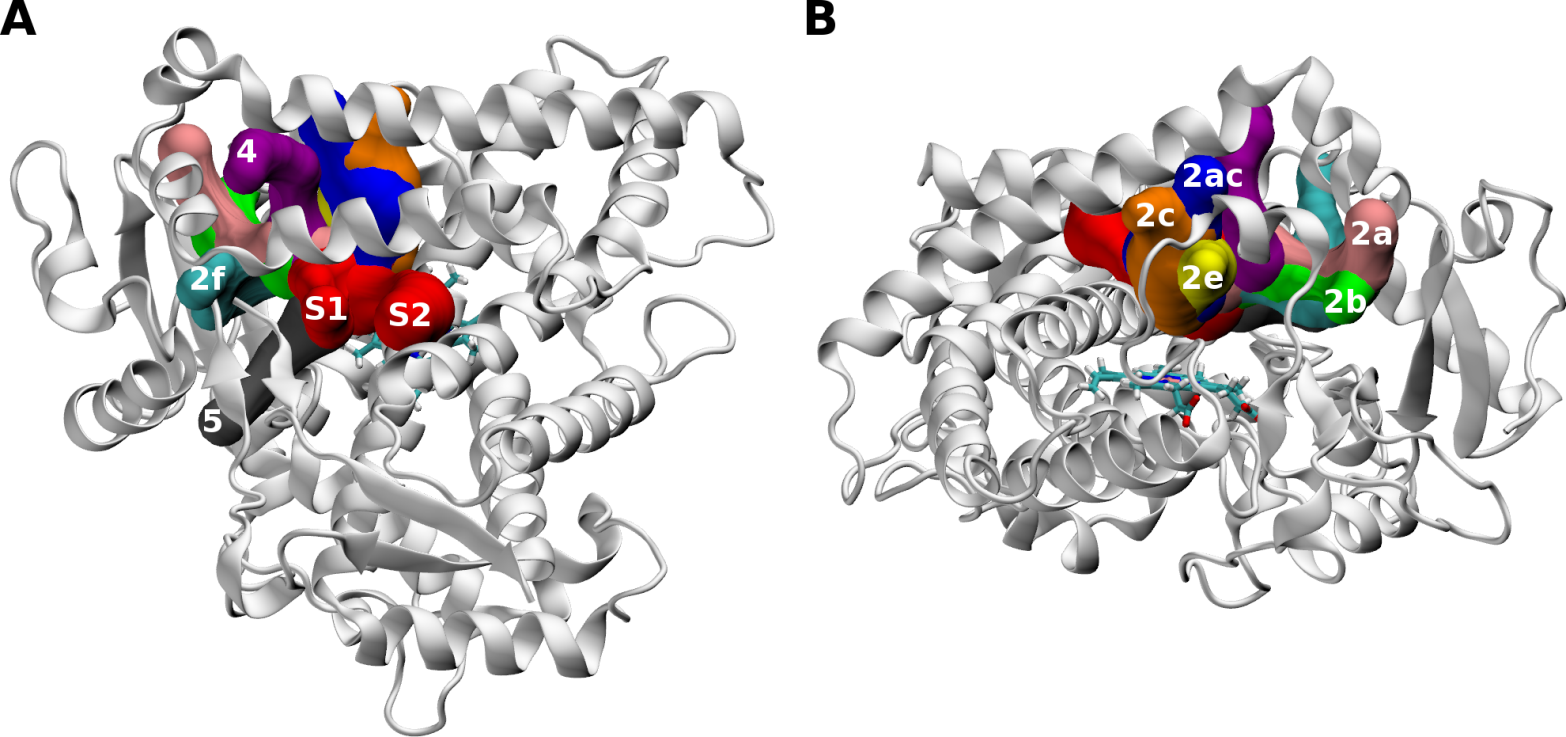
CYP2C9 channels identified during the MD simulations. The channels are labelled according to Cojocaru et al. [26]. The protein is represented in white cartoon and the heme is represented in sticks.

We explored the structure and dynamics of the substrate access channels for the WT and the A477T mutant. Significant changes of the substrate access channels were found to occur in the presence of the mutation A477T. The open channels detected along all MD simulations for the WT and the mutant proteins are summarized in Table 1. CYP2C9 channels were previously studied [26, 27] and our predicted channels were labeled accordingly (see Fig 6A and 6B). We identified similar channels as the previously described ones, except for the so-called solvent channel S, which was found here to be divided into two independent channels named S1 and S2. In all cases, the solvent channel S1 remained more open than S2.

**Table 1:** Percentage of CYP2C9 channels opened during the MD simulations of the WT and the A477T mutant in the apo, diclofenac- and losartan-bound states.

Simulation system	2a	2b	2c	2ac	2e	2f	4	5	S1	S2
WT apo	17.0	32.1	5.9	0.6	34.9	14.2	0.7	13.8	79.4	31.3
A477T apo	20.4	21.7	6.8	0.1	33.6	9.1	0.3	8.0	67.8	30.1
WT diclofenac	40.7	25.0	10.9	1.9	23.6	17.4	2.3	21.2	67.5	33.1
A477T diclofenac	24.5	23.8	3.7	14.6	20.6	37.7	0.3	15.5	65.8	28.5
WT losartan	6.2	5.9	45.6	0.0	12.6	6.1	1.6	8.0	71.7	12.1
A477T losartan	16.3	21.3	49.7	0.0	5.7	12.5	0.1	10.3	60.5	22.6

We observed that several channels were open in more than 20% of the MD simulations of the diclofenac-bound state (2a, 2b, 2e 2f, 5, S1 and S2). This observation is in accordance with the large movements of diclofenac observed during the MD simulations suggesting a dynamic behavior of the channels. Similar results have been reported in a previous study [27] for CYP2C9 bound to flurbiprofen, a NSAID similar to diclofenac. On the contrary, the large losartan molecule showing a very stable position in the binding pocket during the MD simulations triggered a very different behavior regarding the opening and closing of the channels. In fact, only three channels (2c, S1 and S2) were open during the MD simulations of losartan-bound CYP2C9 in accordance with the two structures of the WT and A477T mutant co-crystalized with losartan, PDB ID 5XXI and 5X23 [13], respectively. Interestingly, channel 2c was open in the losartan-bound structures, but closed in all other studied CYP2C9 states. Recently, it has been suggested that the peripheral site situated nearby channel 4 accommodating losartan and palmitic acid molecules in CYP2C9 and CYP2C8, respectively, could play some role in the substrate recognition [13]. However, our analyses indicated that channel 4 was closed in the MD simulations for all WT and mutant protein structures, either in apo or substrate-bound states. Thus, it is not likely this area to be a preferred access channel site.

The comparison between the apo WT and mutant proteins showed several important changes due to the mutation. Most of the substrate and solvent access channels were more closed in the A477T mutant as compared to the WT. More precisely, the channels 2b, 2f, 5 and S1 were more closed in the apo state of the mutant than of the WT (see Table 1). The channel 5 is partly formed by the β turn 4, where the mutation is located. As described above, this protein region changed its conformation in presence of the mutation that may lead to the closing of channel 5. The channel 2b is located far from the mutation residue 477 (> 10 Å). However, the movement of F476 toward L361, L362 and L366 belonging to SRS5 in the A477T mutant structure, as discussed above, likely alters the 2b formation depending of the SRS5 conformation. In addition, the shift of F476 toward L208, as explained above, favorites the closing of the channel S1 in presence of the mutation A477T. All together the channels accessibility results suggest that the F476 shift in the apo A477T mutant plays a critical role in the channel dynamics.

### Substrate docking into CYP2C9 WT and A477T structures

We carried out molecular docking calculations to identify different conformations of the WT and A477T mutant of CYP2C9 properly accommodating the substrates for the catalytic reaction. In order to explore the binding spectrum of CYP2C9, we studied five drugs metabolized by CYP2C9 by molecular docking: diclofenac, flurbiprofen, warfarin, losartan and glimepiride. It has been shown that the metabolism of diclofenac, losartan and glimepiride is strongly affected in the A477T mutant [9–11]. To take into account the plasticity of the CYP2C9 binding pocket we studied multiple conformations of the WT and mutant protein generated by the MD simulations. To avoid redundancy in docking experiments we focused on the centroids structures generated by structural clustering of the binding site over the MD simulations. The most representative 40 centroids (corresponding to clusters covering at least 85% of the MD simulations, S4 Fig) generated for each of the six studied systems (WT apo, WT diclofenac-bound, WT losartan-bound, A477T apo, A477T diclofenac-bound, A477T losartan-bound) were used for docking. In total, the five drug substrates were docked into 5 crystal and 240 CYP2C9 WT/mutant conformations (see S8 Fig).

**Fig 7:**
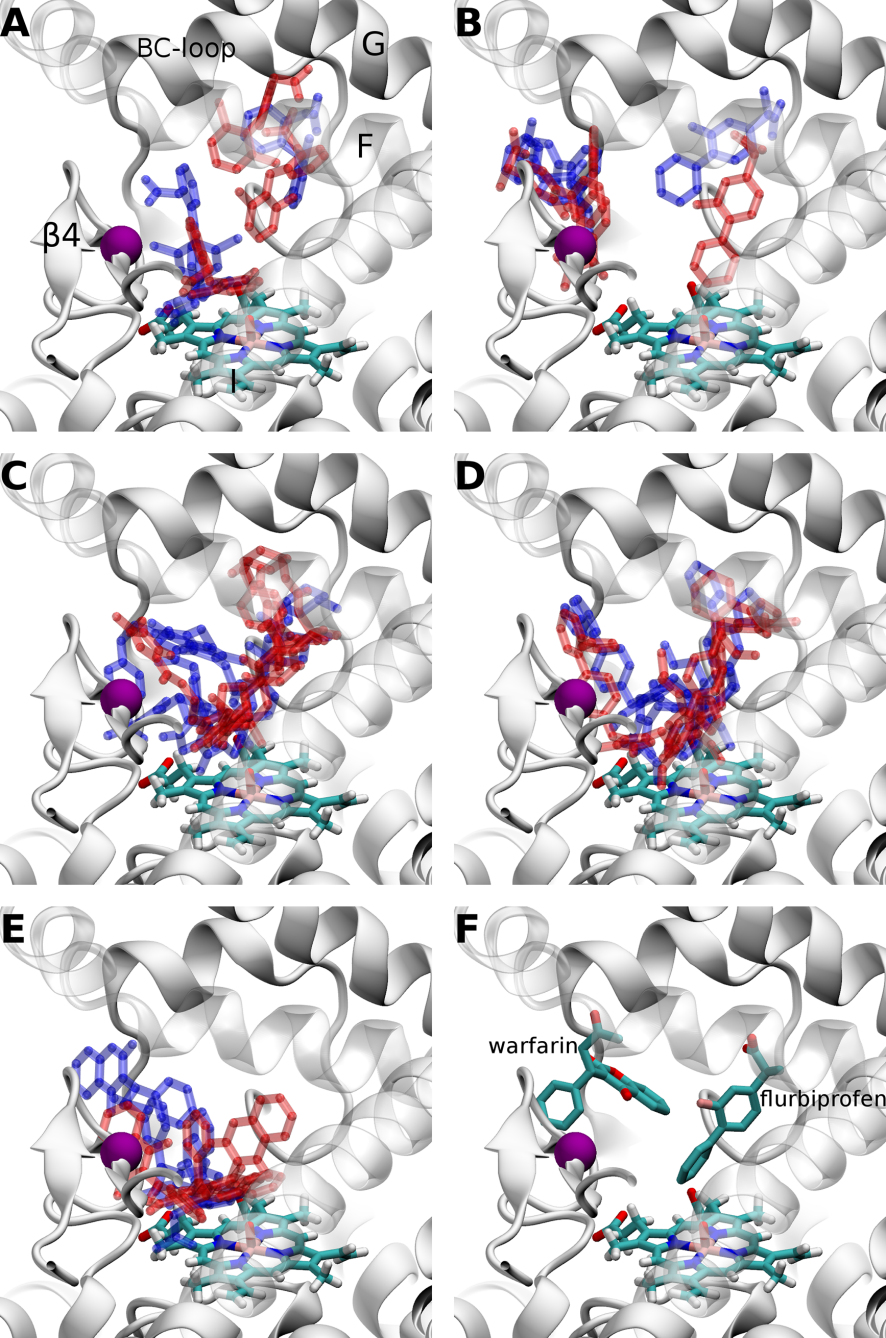
The most representative three docking positions obtained by structural clustering with RMSD of 4 Å over each substrate. The ligands are represented as blue sticks for WT and red sticks for A477T mutant. (A) diclofenac, (B) flurbiprofen, (C) glimepiride, (D) losartan and (E) warfarin. (F) The experimentally known positions of flurbiprofen and warfarin, both represented in sticks. CYP2C9 is represented in white cartoon, Cpd I in sticks and A/T477 residue is represented by a purple sphere.

Ten independent docking calculations were performed for each protein centroid structure. The three top-scored substrate poses were taken for the analysis. This resulted in 360 substrate poses in MD generated WT CYP2C9 conformations and 360 poses in MD generated A477T mutant conformations for each of the five drugs. The two small NSAID flurbiprofen and diclofenac were found to bind in different positions in the CYP2C9 binding pocket, while the other ligands showed preferable binding zones. The comparison of the WT and the mutant showed differences for the diclofenac and flurbiprofen docking in the so called "flurbiprofen-binding" subpocket corresponding to the catalytic site subpocket close to the heme (Fig 7A and 7B). These two ligands were accommodated closer to the helix I (SRS4) in the mutant structures compared to the WT ones. The large glimepiride and losartan molecules were oriented toward the Cpd I oxygen atom and no important differences were detected for their docking positions when comparing the WT and the mutant structures (Fig 7C and 7D). Interestingly, the warfarin was found to bind preferably in the "warfarin-binding" sub-pocket corresponding to the access channel and to an intermediate binding state before reaching a position for the catalysis (Fig 7E and 7F). The warfarin also changed its docking positions in the mutant structures moving toward helix I as it was observed for diclofenac and flurbiprofen.

Then, we focused on the SOM positions regarding the Cpd I oxygen atom, which was included in the docking calculations. We considered that the docking poses showing a distance between the SOM (Fig 1) and the Cpd I catalytic oxygen atom < 6 Å corresponded to a substrate binding mode appropriate for the catalysis (see S8 Fig and Table 2). As seen in S8 Fig, docking of substrates only into the crystal structures of CYP2C9 is not sufficient to explore the binding behavior of the substrates. We did not observe significant differences between the WT and the mutant regarding the number of acceptable poses. However, noticeable differences between the WT and the A477T mutant were found (see Table 2) when focusing on docking poses obtained in the highly representative centroids of the MD simulations (representativity > 6% of the MD simulations). A larger number of acceptable poses for the catalytic reaction were obtained for the WT as compared to the mutant for the most representative centroids. These results indicated that some conformations of the A477T mutant were still able to accommodate the substrates, however, mutant conformations that properly bound the substrates were visited for a shorter period of time of the MD simulations compared to the WT. The detailed analysis showed that the 3 top-scored docking poses with distance < 6 Å between the SOM and the Cpd I oxygen were obtained in: 3 centroids (cluster numbers 1, 2 and 3) generated by WT apo MD simulations; 2 centroids (cluster numbers 0 and 1) generated by WT diclofenac-bound MD simulations; 3 centroids (cluster numbers 0, 1 and 2) generated by WT losartan-bound MD simulations, 1 centroid (cluster number 0) generated by A477T apo MD simulations; 2 centroids (cluster numbers 0 and 1) generated by A477T diclofenac-bound MD simulations; 3 centroids (cluster numbers 0, 1 and 2) generated by A477T losartan-bound MD simulations. They represented 25.76%, 19.64%, 24.98%, 8.44%, 20.31% and 23.36% of the entire 250 ns MD simulations for each of the six studied systems, respectively (see S4A Fig and S1 Table). We should note that only 1 centroid structure suitable for substrate docking was identified from the A477T apo mutant MD simulations, thus confirming the altering effect of the mutation on the binding site conformation. As observed from the MD RMSF analysis, the centroid conformations generated by the MD simulations in presence of a bound substrate were less affected by the mutation regarding the substrate docking. In total, 8 WT and 6 mutant centroids (noted here as “best" centroids) were identified to properly accommodate the substrates with distance < 6 Å between the SOM and the Cpd I oxygen atom required for the catalytic reaction.

**Table 2:** Number of the top 3 scored docking poses within a distance of 6 Å between the SOM and the Cpd I oxygen docked in 120 WT and 120 A477T structures corresponding to the 40 MD centroids for each MD case. The centroids showing > 6% representativity correspond to a cluster covering at least 6% of the conformers generated along the 250 ns MD simulations.

Substrate	N^o^ of poses into 120 WT centroids	N^o^ of poses into 120 A477T centroids	N^o^ of poses into WT centroids showing > 6% representativity	N^o^ of poses into A477T centroids showing > 6% representativity
Diclofenac	85	83	8	5
Flurbiprofen	92	102	12	7
Glimepiride	63	72	7	2
Losartan	83	89	7	3
Warfarin	86	77	10	4

In order to analyze other possible positions of the losartan in the CYP2C9 active site we compared the docking poses of losartan with the recently reported co-crystalized structures, PDB ID 5XXI (WT–losartan bound) and 5X23 (A477T mutant–losartan bound). The most similar positions were found when the losartan was docked into the active site of the centroid number 4 of CYP2C9 WT apo representing 5.76% of the entire MD simulations for WT apo (RMSD = 5.41 Å) and of the centroid number 0 of A477T mutant–losartan bound representing 6.59% of the entire MD simulations for the A477T apo mutant–losartan bound (RMSD = 4.36 Å). These docking poses suggest that losartan can also adopt positions similar to the co-crystalized ones in CYP2C9 WT and CYP2C9 A477T mutant–losartan bound. Although RMSD are not very low, the losartan is similarly oriented in the docked and experimental structures (see S9 Fig). However, such positions are not consistent with the hydroxylation catalytic reaction.

**Fig 8:**
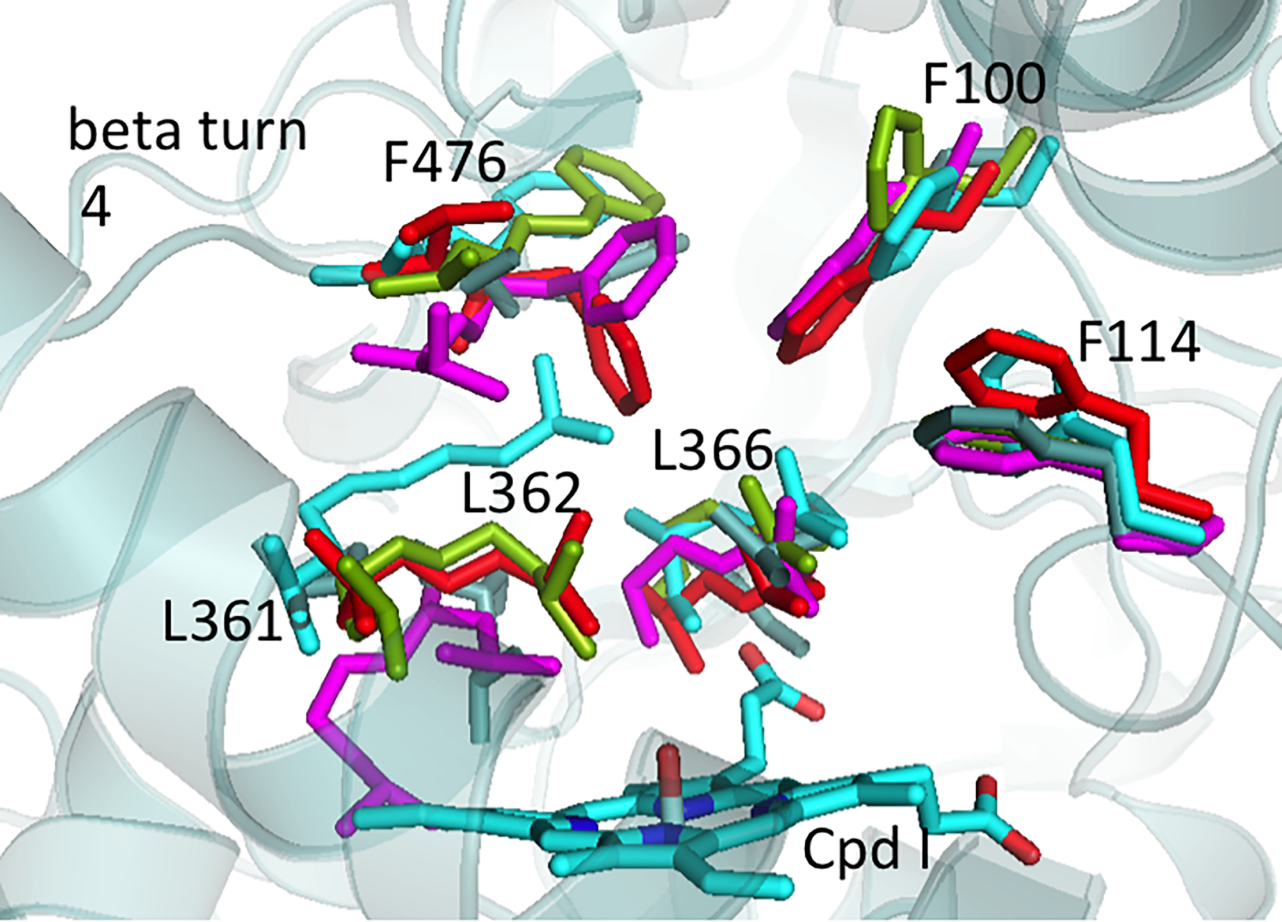
Key residues of the active site of the best identified CYP2C9 structures for substrate docking generated from MD simulations of diclofenac-bound CYP2C9. The side chains of the diclofenac-bound WT structures are colored in grey (centroid 0), cyan (centroid 1), light green (centroid 2). The side chains of the diclofenac-bound A477T structures are colored in red (centroid 0) and violet (centroid 1).

In the three best WT apo MD derived centroids the residues F476 and L362 adopted different conformations (Fig 8). The residue L366 kept its hydrophobic contacts with the heme as in the apo X-ray structures. On the contrary, L366 was observed to shift far from the heme in the best apo mutant centroid. In the best centroids derived from the WT diclofenac-bound state MD simulations L361, L362, F100 and F114 show different orientations in the binding pocket due to the large motions of the diclofenac during the MD simulations. Compared to the WT diclofenac-bound MD based centroids, the mutant diclofenac-bound MD based centroids showed very important shift of F100 and the β turn 4 containing F476 (see Fig 8). In the centroids generated from the MD simulations of the WT and A477T mutant losartan-bound states, L362 adopted different conformations. Overall the above cited residues showed large mobility and adopted different conformations depending on the presence of the mutation A477T or a bound substrate during the MD simulations.

## Conclusions

We investigated molecular mechanisms implicated in the altered substrate metabolism in CYP2C9.30 exhibiting < 50% of the WT enzyme activity. MD simulations performed for the active species of the enzyme, Compound I, in apo or substrate-bound states, and binding energy analyses for several drugs revealed molecular mechanisms involved in the defective drug metabolism in carriers of CYP2C9*30. Our data demonstrated that the A477T mutation present in CYP2C9.30 led to increased rigidity of the SRS1 and SRS5 regions and to shifting of the β turn 4 of SRS6 toward helix F thus decreasing the substrate access to some protein channels. We observed different behaviors of the substrates diclofenac and losartan during the MD simulations for the WT and the A477T mutant, suggesting that A477T can alter differently the metabolism of various substrates. The overall stabilization of the WT and the mutant structures in the cases of a bound substrate observed for the performed here MD simulations as well as for the available crystal structures demonstrated the importance to study structural and dynamics properties of the apo protein in order to detect molecular mechanisms due to the mutation without screening effects of a bound ligand. Molecular docking of five drug substrates of CYP2C9 into WT and A477T mutant structures generated by MD simulations indicated that some conformations of the A477T mutant were able to correctly accommodate the substrates, however, their number was very low compared to the WT MD conformations. All together these conformational and dynamic changes are believed to be involved in the governing mechanism of the catalytic activity dysfunction. Finally, an ensemble of representative conformations of the WT and A477T mutant properly accommodating the substrates were identified, these structures can be used for future prediction of unknown CYP2C9.1 and CYP2C9.30 substrates and drug-drug interactions.

## Materials and methods

### System preparation

Six crystallographic structures of CYP2C9 were taken from the Protein Data Bank (PDB) and analysed: the native sequence flurbiprofen–bound one (PDB ID 1R9O) [28], losartan-bound one (PDB ID 5XXI [13]), losartan-bound CYP2C9.30 (containing the mutation A477T) (PDB ID 5X23 [13]), and three structures of CYP2C9 containing the mutations K206E, I215V, C216Y, S220P, P221A, I222L, I223L: the apo CYP2C9 one (PDB ID 1OG2 [25]), the warfarin-bound one (PDB ID 1OG5 [25] and the inhibitor 2QJ-bound one (PDB ID 4NZ2 [29]. We decided to construct our initial model of CYP2C9 based on the only one available apo crystal structure of CYP2C9 (PDB ID 1OG2) in order to getaway possible effects of bound ligands on the initial enzyme structure. We restored the 7 original CYP2C9 residues mutated in the crystallized enzyme. In addition, the side-chain orientation of R108 was rotated in order to permit its hydrogen bond formation with bound substrates. The pKa values of the titratable groups of the CYP2C9 WT and A477T mutant proteins were calculated with the FDPB approach using the web server PCE [30]. The hydrogen atoms were then added using the DockPrep utility of Chimera [31]. In order to check the effects of the 7 mutations present in the crystallized apo 1OG2 structure on our starting model conformation of the CYP2C9 WT, we performed initial molecular dynamics (MD) simulations of the crystallized apo 1OG2 structure containing these mutations and the apo 1OG2 structure with restored native CYP2C9 residues. The same MD protocol of 250 ns used for the other MD simulations and described in the next section was employed. The analysis has shown that the mutations present in 1OG2 and 1OG5 do not critically affect the overall protein dynamics and structure neither the dynamic behaviour of the BC loop and the β turn 4 shown in this study to be affected by the A477T mutation in CYP2C.30.

The initial models of diclofenac-bound and losartan-bound WT and A477T mutant structures were generated using docking with Autodock 4.2 program [32]. The best substrate conformations showing the lowest binding energies and a correct position of SOM (see Fig 1) (i.e. SOM within distance of 6 Å to the heme catalytic oxygen) were chosen as starting conformations for MD simulations.

### Molecular dynamics simulations

The six initial CYP2C9 models (WT apo, A477T apo, WT diclofenac-bound, A477T diclofenac-bound, WT losartan-bound, A477T losartan-bound) were fully solvated with explicit TIP3P water molecules [33] and neutralized resulting in systems of about 60,000 atoms. The CHARMM36 force field was used [34]. The CGenFF force field [35] was used to parameterize diclofenac with acceptable penalty score [36]. In contrary, very high penalty score (> 205) were obtained for losartan using CGenFF. Thus, we performed quantum-mechanical calculations by means of density functional theory (DFT) to obtain the losartan parameters. Geometry optimization was performed by using B3LYP functional and 6–31g(d) basis set. The obtained losartan structure was located at a minimum of the potential energy surface through frequency calculations at the same level of energy (see S10 Fig). Atomic charges derived from the electrostatic potential were calculated using the ChelpG scheme. All calculations were performed with the Gaussian 09 software (version C.01, Gaussian, Inc., Wallingford CT, 2010). Atomic parameters of losartan obtained with quantum mechanical calculations are given in S2 and S3 Tables. The force field for the heme Cpd I was taken from [37].

The NAMD2.6 program [38] was used for minimization and MD simulations. All systems containing water and ion molecules were firstly minimized with 5000 steps of conjugate gradient to remove poor contacts and relax the system. The systems were then submitted to a 1 ns MD simulation in order to equilibrate water and ion molecules located in the vicinity of the protein while the protein atoms were kept fixed. In a second stage, all constraints were removed and 5 MD production runs, each of 50 ns, were executed for each of the six CYP2C9 systems. Velocities were randomly assigned with different seeds for each trajectory. The particle mesh Ewald [39] method was used for electrostatic interactions, and the van der Waals interactions were computed with a switching function applied in the range 9–10 Å. The NPT ensemble was used (1.01325 bar and 300 K) with Langevin dynamics and a Nosé–Hoover–Langevin piston pressure control [40]. The integration step was set to 1 fs. The conformations were saved every 5 ps, resulting in 10,000 conformations per MD trajectory. The MD simulations were performed for 250 ns for each of the six studied systems. To probe the impact of longer MD simulations, we run an additional single MD simulation of 200 ns for the A477T mutant apo. Interestingly, the single MD simulation of 200 ns explored much less conformational space than the combined 5x50ns MD simulations and the conformational space explored by the single 200 ns run was covered by the 5 x50 ns MD simulations for the CYP2C9 A477T mutant (see S4 Fig).

### Representative structures of the substrate binding pocket

To find the most representative structures of the substrate binding pocket for each of the six systems we merged the five MD trajectories. For the structural clustering, we took 25,000 protein conformations (1 conformation every 10 ps) for each system and used the quality threshold algorithm [41] of the VMD software [42]. Following our previous protocols for multiple receptor ensemble generation [43, 44], a Root Mean Square Deviation (RMSD) distance of at least 1.0Å for all atoms of the binding site was chosen as the clustering criteria. The following residues were considered as part of the enzyme binding pocket: I99, F100, L102, A103, A106, N107, R108, V113, F114, L201, N204, I205, I207, L208, S209, K232, L233, L234, A291, V292, D293, F295, G296, A297, G298, T299, E300, T301, L366, F476 and A(T)477. We took the centroid structure of each cluster in order to define a representative set of protein conformations for subsequent analysis.

### Substrate docking

Five drugs known to be CYP2C9 substrates were docked into the crystal CYP2C9 structures PDB IDs: 1OG5 and 4NZ2 with restored native residues, 1R9O, 5XXI, 5X23, as well as into the initial models of the WT or the mutant A477T and into 240 representative conformations selected after the MD simulations for the six studied systems. The heme was kept in the intermediate Cpd I state. Drug protonation states were calculated at pH 7.4 with the major microspecies option of the ChemAxon package (ChemAxon www.chemaxon.com). Interestingly, ChemAxon predicted two different pKa values for losartan, 4.29 or 8.0, starting from two different smile structures downloaded from BindingDB and DrugBank, respectively. We analyzed possible protonation of the losartan molecules co-crystallized with CYP2C9 in the PDB structures ID 5X23 and 5XXI and decided to consider the protonated state of the losartan, which corresponds better to the losartan environment in the binding sites. Gasteiger atom charges were added using the AutoDockTools package. Molecular dockings were carried out with the Autodock 4.2 program [32]. A grid spacing of 0.375 Å was used. The search space was centered on the protein binding sites with a box size of 24x25x30 Å. Ten independent docking runs were performed using the Lamarckian genetic algorithm [45] with an initial population of 150 random individuals and a maximum number of 25,000,000 energy evaluations. During docking, ligand molecules were flexible while the protein was kept rigid.

### Channel dynamics determining

The channel dynamics for the WT and the A477T mutant were investigated with the CAVER software [46]. All CYP2C9 conformations generated from the MD simulations were analyzed using CAVER (50,000 for each system). A probe radius of 1.2 Å was used for channel prediction. The starting point was set at the center of the binding pocket. In case of a bound substrate during the MD simulations, the ligand was removed prior to channel searching.

## Supporting information

S1 Fig 3D structure of CYP2C9. (PDF)

S2 Fig Root Mean Square Deviations of all heavy atoms for each MD simulation. (PDF)

S3 Fig RMSF differences of Ca atoms between the WT and A477T proteins for the apo, diclofenac bound and losartan bound states. (PDF)

S4 Fig Structural clustering of the MD generated conformers. (PDF)

S5 Fig Hydrophobic contacts between F476 and other residues of the CYP2C9 binding pocket monitored over the MD simulations. (PDF)

S6 Fig Initial and preferential positions of losartan in the CYP2C9 active site during the MD simulations of the WT and A477T variant losartan-bound systems. (PDF)

S7 Fig RMSD of Compound I during the MD simulations. (PDF)

S8 Fig Docking scores of docking poses as a function of distance between the catalytic oxygen of Cpd I and SOM. (PDF)

S9 Fig Docking poses of losartan similar to the co-crystallized structures. (PDF)

S10 Fig Molecular structure of losartan as obtained with quantum-mechanical geometry optimization. (PDF)

S1 Table Centroid structures with representativity ≥ 6% along the MD simulations allowing to dock the substrates with distances between SOM and Cpd I catalytic oxygen within 6 Å. (PDF)

S2 Table Atomic charges of losartan used as parameters for the molecular dynamics simulations. (PDF)

S3 Table Atomic parameters of losartan obtained with quantum mechanical calculations. (PDF)
