# Supplementary material for: Insights into molecular mechanisms of drug metabolism dysfunction of human CYP2C9*30
Source: PLoS One. 2018 May 10;13(5):e0197249. doi: 10.1371/journal.pone.0197249 (PMC5944999; doi:10.1371/journal.pone.0197249)
Supplement: S1 Fig — (PDF) [file pone.0197249.s001.pdf]

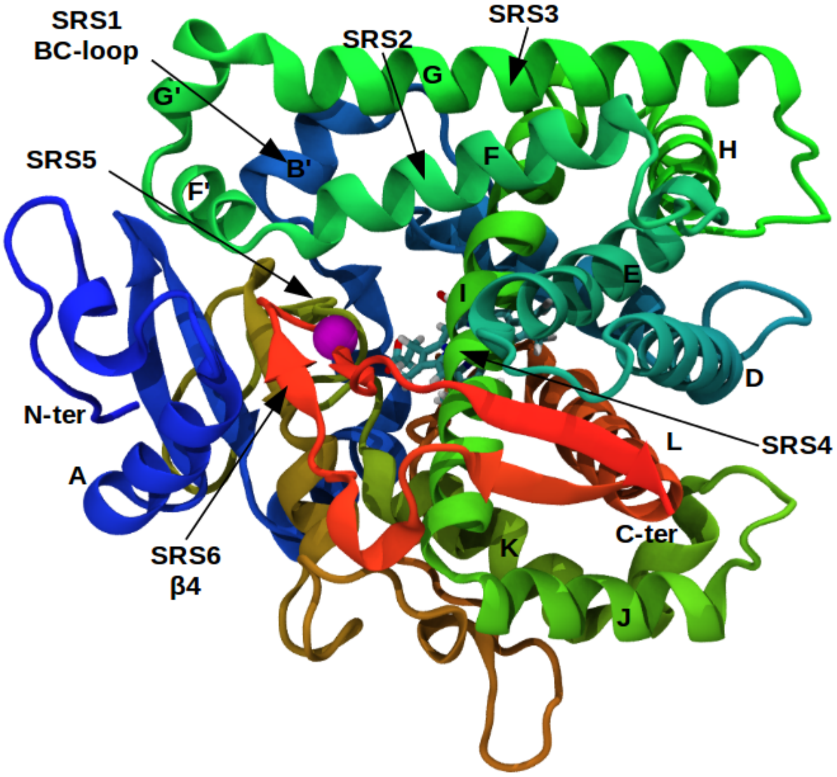

Figure S1. 3D structure of CYP2C9, PDB ID: 1OG5. The protein is represented as cartoon. α-helices are labelled according to the standard CYP nomenclature. The mutation residue A477T is shown as a purple sphere. The Substrate Recognition Sites (SRS) are also noted for orientation. The heme is shown in a stick representation.
