# Supplementary material for: Insights into molecular mechanisms of drug metabolism dysfunction of human CYP2C9*30
Source: PLoS One. 2018 May 10;13(5):e0197249. doi: 10.1371/journal.pone.0197249 (PMC5944999; doi:10.1371/journal.pone.0197249)
Supplement: S2 Fig — (PDF) [file pone.0197249.s002.pdf]

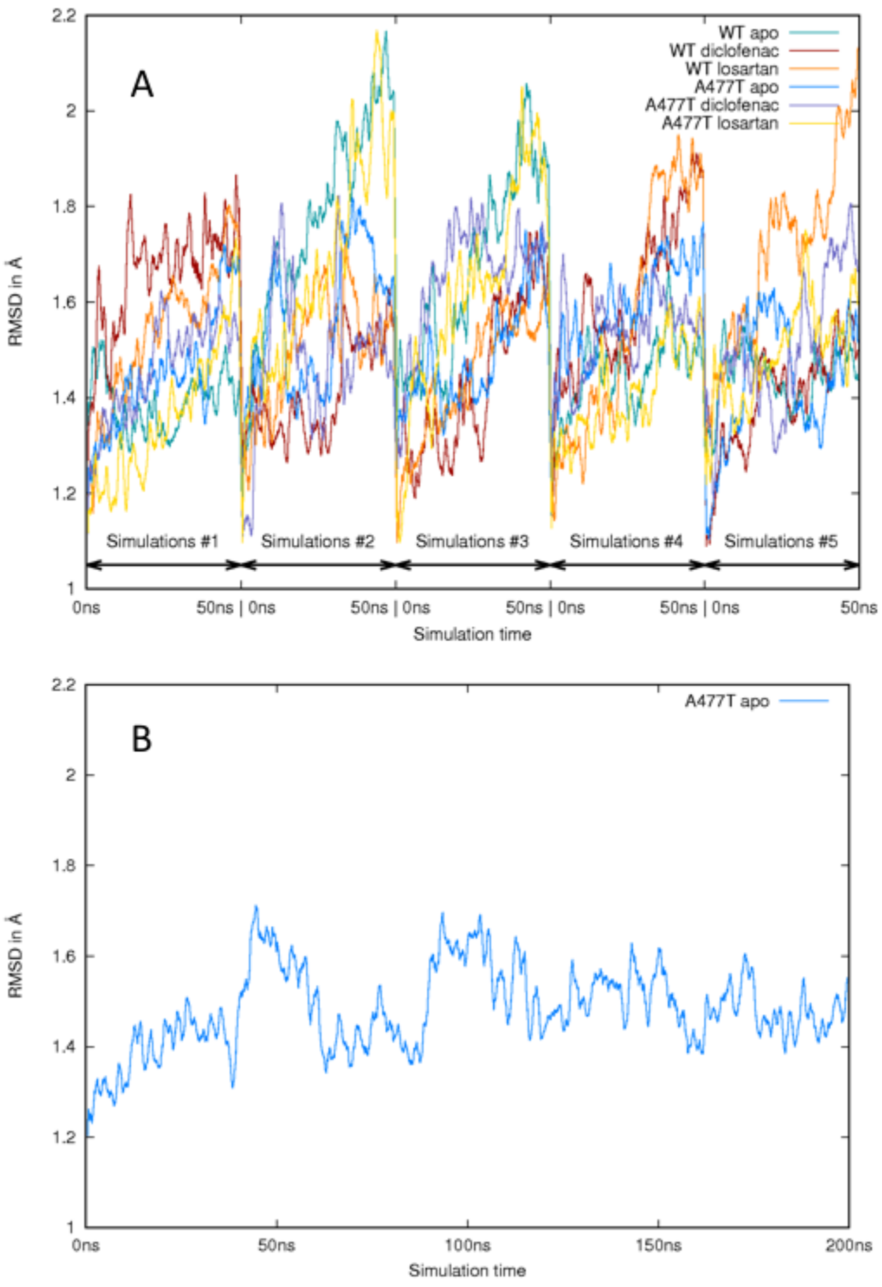

Figure S2. (A). Root Mean Square Deviations of backbone atoms for each MD simulation of 50 ns. (B). Root Mean Square Deviations of backbone atoms for the MD simulation of 200 ns for CYP2C9 A477T apo.
