# Supplementary material for: Insights into molecular mechanisms of drug metabolism dysfunction of human CYP2C9*30
Source: PLoS One. 2018 May 10;13(5):e0197249. doi: 10.1371/journal.pone.0197249 (PMC5944999; doi:10.1371/journal.pone.0197249)
Supplement: S3 Fig — (PDF) [file pone.0197249.s003.pdf]

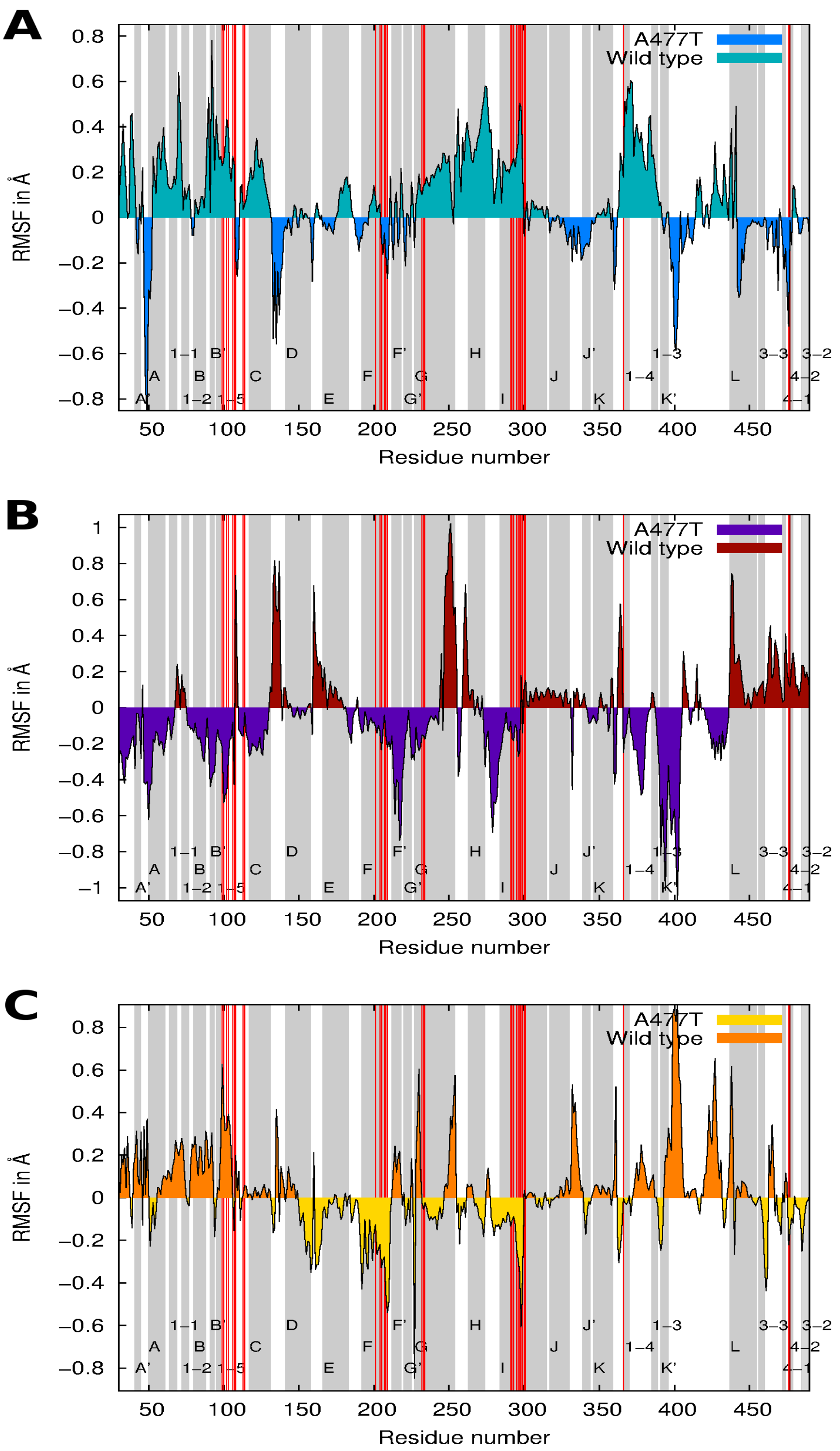

Figure S3. RMSF differences of Cα atoms between the WT and A477T proteins for the apo (A), diclofenac bound (B) and losartan bound (C) states. Positive values represent higher fluctuations for the WT whereas negative values represent higher fluctuations for the mutant. Secondary structures are labelled (e.g. helices A, B, etc; beta-sheets 1-1, 1-2, etc.) and depicted as grey areas. Residues involved in the binding pocket are noted by red lines.
