# Supplementary material for: Insights into molecular mechanisms of drug metabolism dysfunction of human CYP2C9*30
Source: PLoS One. 2018 May 10;13(5):e0197249. doi: 10.1371/journal.pone.0197249 (PMC5944999; doi:10.1371/journal.pone.0197249)
Supplement: S4 Fig — (PDF) [file pone.0197249.s004.pdf]

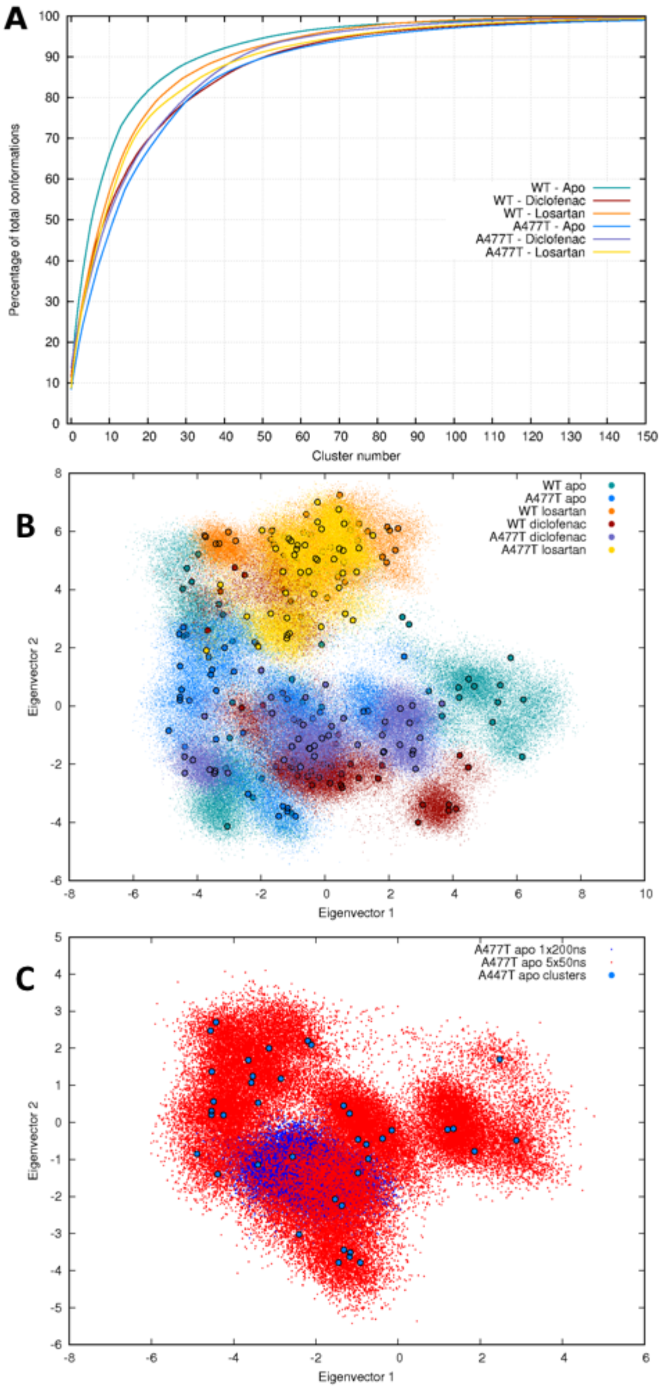

Figure S4. Structural clustering of the MD generated conformers. (A) Percentage of total number of conformations corresponding to the generated clusters of the concatenated MD trajectories 5x50ns. (B) Projection on the first and second lowest-frequencies quasi-harmonic modes, obtained from a Quasi Harmonic Analysis of the binding pocket of the concatenated MD trajectories 5x50 ns. Each point represents the projection of each snapshot from the concatenated MD simulations of: WT apo (cyan), mutant apo (blue), WT diclofenac (red), mutant diclofenac (purple), WT losartan (orange) and mutant losartan (yellow). The circles represent the frames corresponding to the 40 most representative centroids. (C) Projection on the first and second lowest-frequencies quasi-harmonic modes, obtained from a Quasi Harmonic Analysis of the binding pocket of the MD trajectories of the mutant apo. Each point represents the projection of each snapshot from the concatenated MD simulations 5x50ns of the mutant apo (red points) and from the 200 ns MD simulation of the mutant apo (blue points). The circles represent the frames corresponding to the 40 most representative centroids of the MD simulations 5x50ns of the mutant apo.
