# Supplementary material for: Insights into molecular mechanisms of drug metabolism dysfunction of human CYP2C9*30
Source: PLoS One. 2018 May 10;13(5):e0197249. doi: 10.1371/journal.pone.0197249 (PMC5944999; doi:10.1371/journal.pone.0197249)
Supplement: S5 Fig — (PDF) [file pone.0197249.s005.pdf]

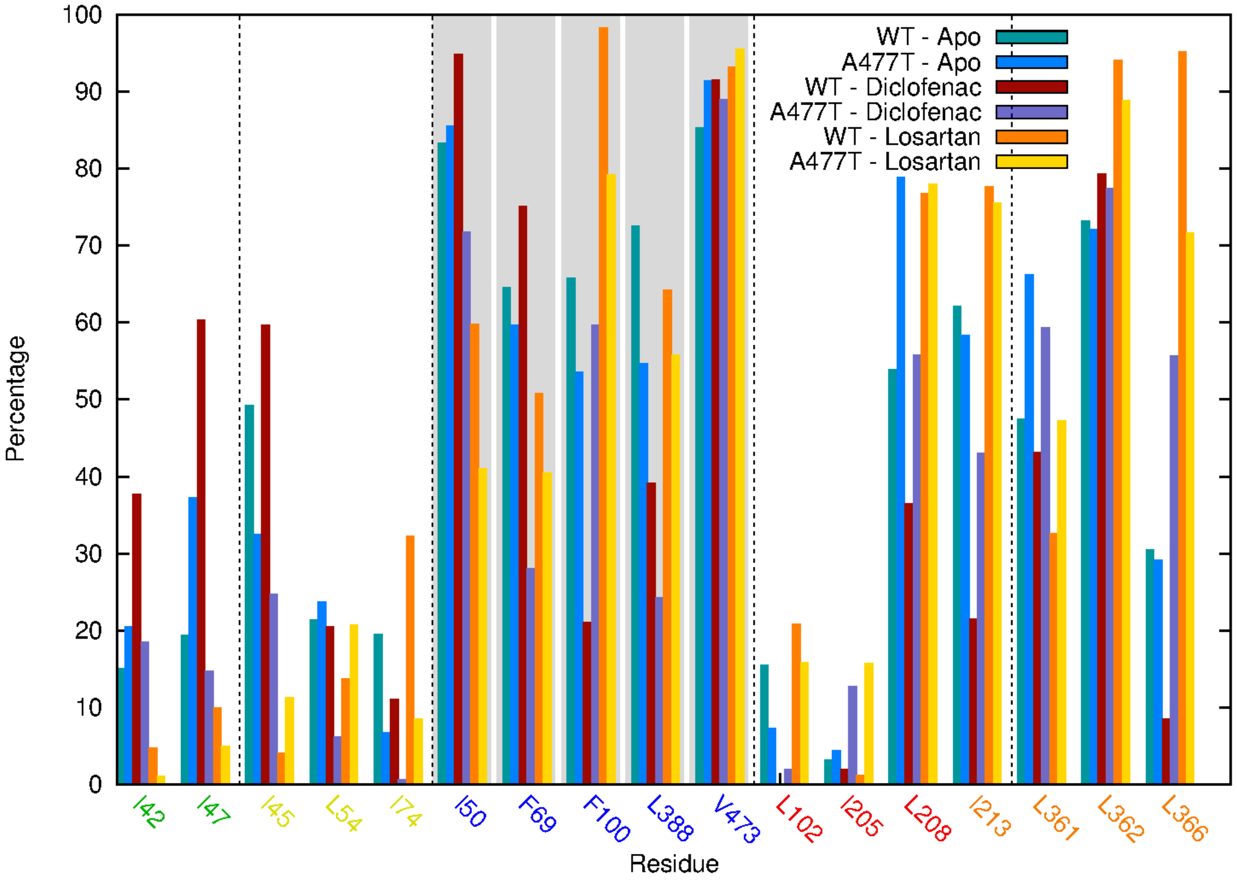

Figure S5. Hydrophobic contacts between F476 and other residues of the CYP2C9 binding pocket monitored over the MD simulations. The histograms represent the percentage of contacts along the concatenated MD simulations. Grey areas represent contacts observed in the 1OG5 X-ray structure.
