# Supplementary material for: Insights into molecular mechanisms of drug metabolism dysfunction of human CYP2C9*30
Source: PLoS One. 2018 May 10;13(5):e0197249. doi: 10.1371/journal.pone.0197249 (PMC5944999; doi:10.1371/journal.pone.0197249)
Supplement: S6 Fig — (PDF) [file pone.0197249.s006.pdf]

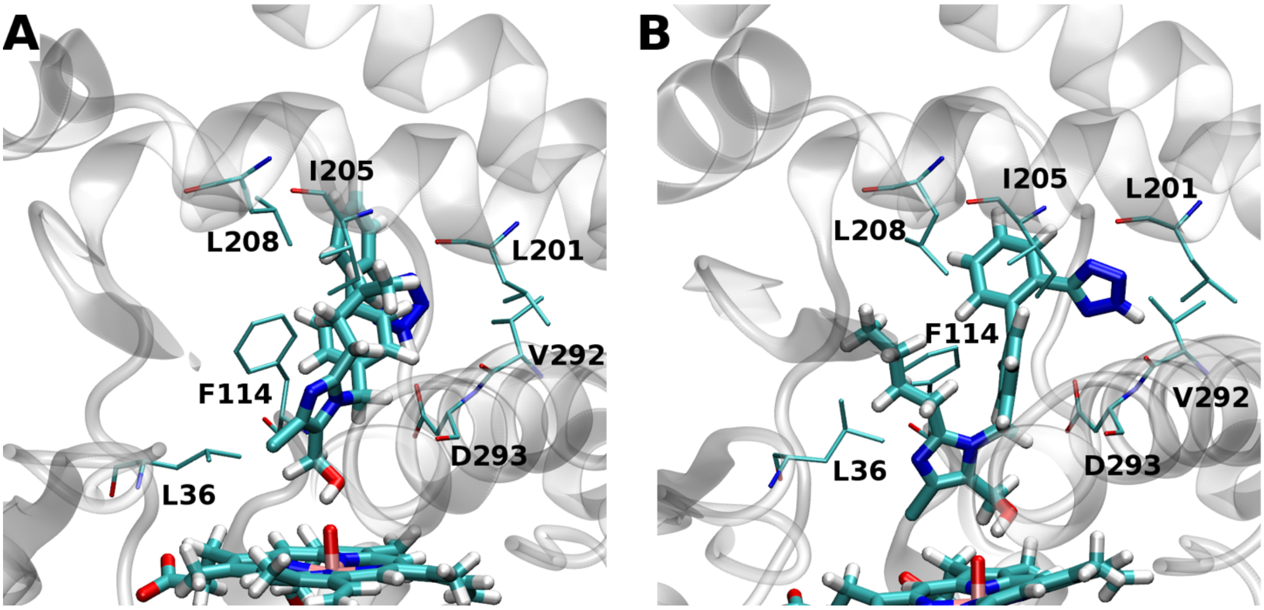

Figure S6. Initial (A) and preferential (B) positions of losartan in the CYP2C9 active site during the MD simulations of the WT and A477T variant losartan-bound systems. The protein is shown as a cartoon in white. The heme, losartan and residues of the protein interacting with losartan are shown in sticks.
