# Supplementary material for: Insights into molecular mechanisms of drug metabolism dysfunction of human CYP2C9*30
Source: PLoS One. 2018 May 10;13(5):e0197249. doi: 10.1371/journal.pone.0197249 (PMC5944999; doi:10.1371/journal.pone.0197249)
Supplement: S7 Fig — (PDF) [file pone.0197249.s007.pdf]

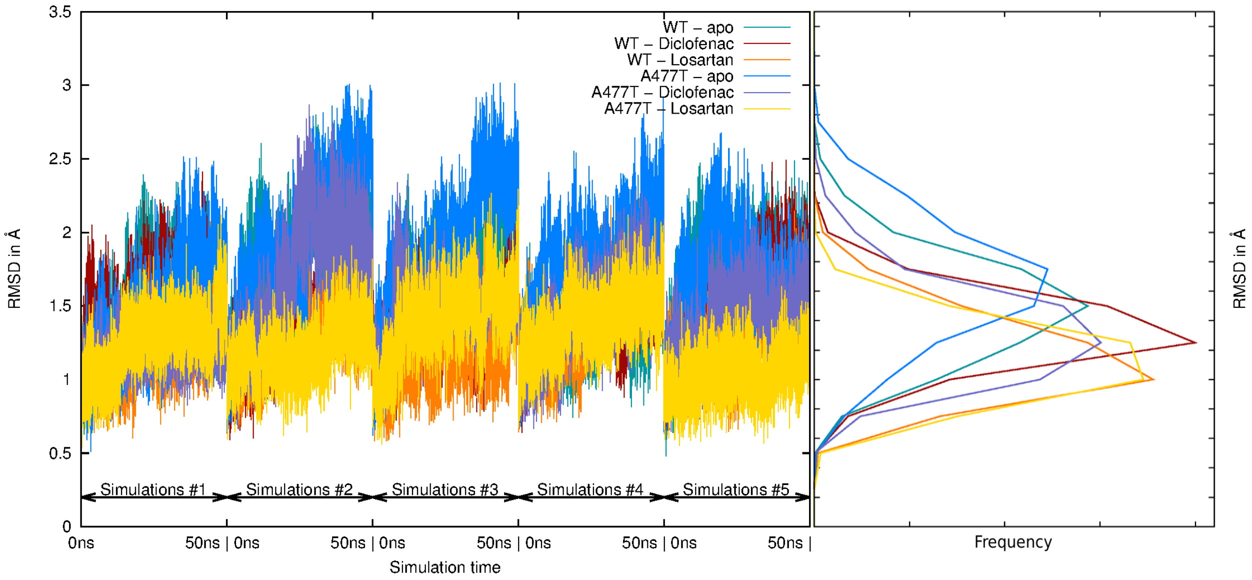

Figure S7. RMSD of Compound I during the MD simulations. The left panel represents the time evolution. The right panel represents the corresponding distributions.
