# Supplementary material for: Insights into molecular mechanisms of drug metabolism dysfunction of human CYP2C9*30
Source: PLoS One. 2018 May 10;13(5):e0197249. doi: 10.1371/journal.pone.0197249 (PMC5944999; doi:10.1371/journal.pone.0197249)
Supplement: S8 Fig — (PDF) [file pone.0197249.s008.pdf]

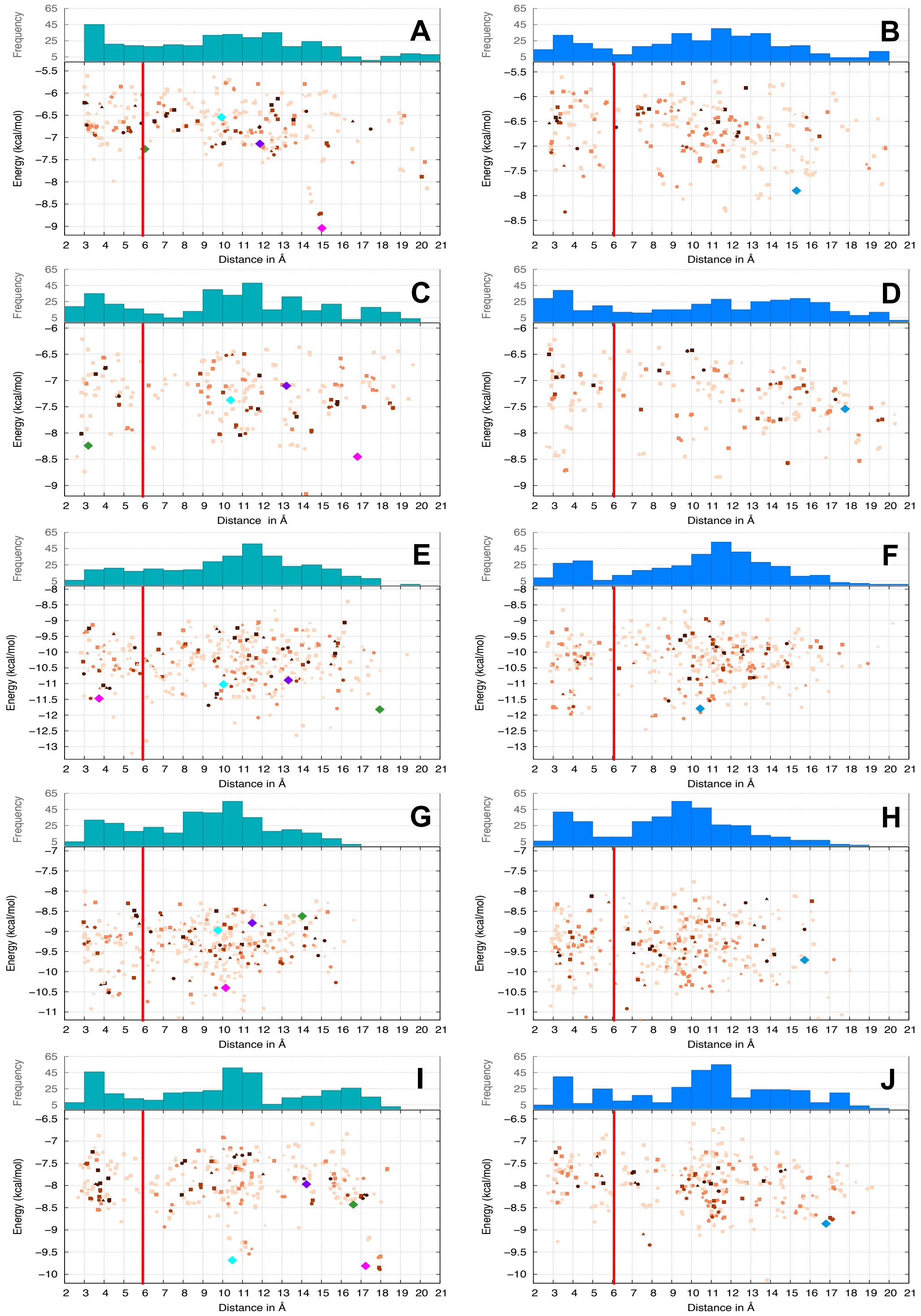

Figure S8. Docking scores of all generated docking poses as a function of distance between the catalytic oxygen of Cpd I and SOM. Docking scores of diclofenac in WT (A) and A477T (B), flurbiprofen in WT (C) and A477T (D), glimepiride in WT (E) and A477T (F), losartan in WT (G) and A477T (H), warfarin in WT (I) and A477T (J) are shown. The points are colored by the representativity of the corresponding centroid structure where the substrate was docked into (light orange < 2%, orange < 4%, brown < 6% and dark brown >6%). Histograms on top represent the number of points for a distance interval of 1 Å. Circle, triangle and square points correspond to the poses ranked as top 1, 2 and 3, respectively. The cyan, violet, green and pink diamonds show the best-energy docking poses into the WT crystal structures of CYP2C9 with restored native residues PBD IDs 1OG5, 4NZ2, and the native WT of CYP2C9 PDB IDs 1R9O and 5XXI, respectively. The blue diamonds show the best-energy docking poses into the mutant CYP2C9.30 PDB ID 5X23.
