# Supplementary material for: Insights into molecular mechanisms of drug metabolism dysfunction of human CYP2C9*30
Source: PLoS One. 2018 May 10;13(5):e0197249. doi: 10.1371/journal.pone.0197249 (PMC5944999; doi:10.1371/journal.pone.0197249)
Supplement: S9 Fig — (PDF) [file pone.0197249.s009.pdf]

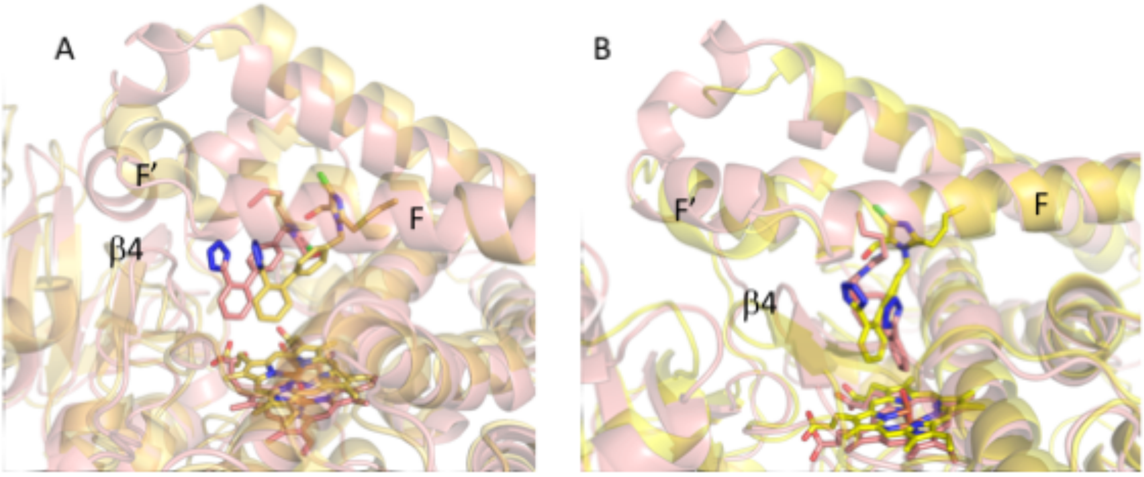

Figure S9. Docking poses of losartan similar to the co-crystallized structures. The yellow cartoon and sticks show the co-crystalized structure of CYP2C9 WT (PDB ID 5XXI) (A) and CYP2C9 A477T (PDB ID 5X23) (B). The salmon cartoon and sticks show the best-energy docking poses of losartan in the centroid number 4 conformation of CYP2C9 WT-apo (A) and the centroid number 0 conformation of CYP2C9 mutant –bound losartan (B).
