# Supplementary material for: Insights into molecular mechanisms of drug metabolism dysfunction of human CYP2C9*30
Source: PLoS One. 2018 May 10;13(5):e0197249. doi: 10.1371/journal.pone.0197249 (PMC5944999; doi:10.1371/journal.pone.0197249)
Supplement: S10 Fig — (PDF) [file pone.0197249.s010.pdf]

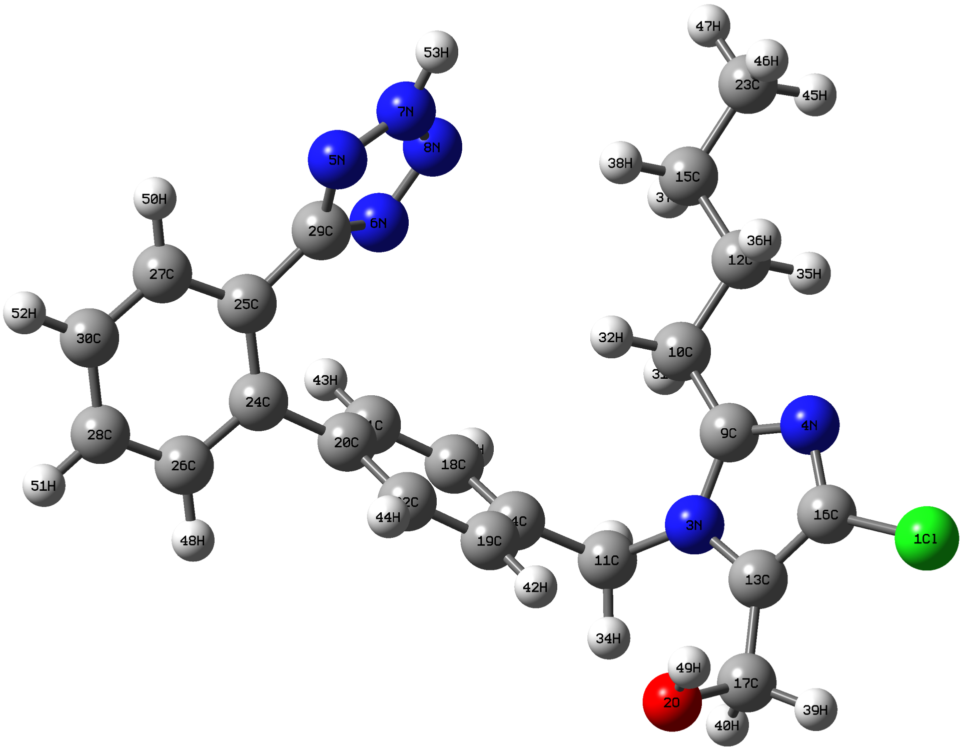

Figure S10. Molecular structure of losartan as obtained with quantum-mechanical geometry optimization.
