# Supplementary material for: Insights into molecular mechanisms of drug metabolism dysfunction of human CYP2C9*30
Source: PLoS One. 2018 May 10;13(5):e0197249. doi: 10.1371/journal.pone.0197249 (PMC5944999; doi:10.1371/journal.pone.0197249)
Supplement: S1 Table — (PDF) [file pone.0197249.s011.pdf]

Table S1. Centroid structures with representativity ≥ 6 % along the MD simulations allowing to dock the substrates with distances between SOM and Cpd I catalytic oxygen within 6 Å.

Docked substrate	N° Centroids (starting from 0) - N° poses - % centroids representativity					
	WT Apo	WT Diclofenac	WT Losartan	A477T Apo	A477T Diclofenac	A477T Losartan
Diclofenac	1, 3 - 4 - 17.93	0 - 2 - 11.58	1 - 2 - 7.56	0 - 1 - 8.44	1 - 1 - 6.59	1 - 3 - 8.48
Flurbiprofen	2, 3 - 6 - 14.75	None	1, 2 - 6 - 14.62	None	1 - 2 - 6.59	0, 2 - 5 - 14.88
Glimepiride	2 - 1 - 7.83	1 - 3 - 8.06	0, 1, 2 - 3 - 24.98	None	None	1 - 2 - 8.48
Losartan	3 - 3 - 6.92	1 - 2 - 8.06	2 - 2 - 7.06	None	0 - 1 - 13.72	2 - 2 - 6.12
Warfarin	1, 3 - 4 - 17.93	0 - 1 - 11.58	0, 1 - 5 - 17.92	None	1 - 2 - 6.59	1 - 2 - 8.48
