# Supplementary material for: Insights into molecular mechanisms of drug metabolism dysfunction of human CYP2C9*30
Source: PLoS One. 2018 May 10;13(5):e0197249. doi: 10.1371/journal.pone.0197249 (PMC5944999; doi:10.1371/journal.pone.0197249)
Supplement: S2 Table — (PDF) [file pone.0197249.s012.pdf]

Table S2. Atomic charges derived from the electrostatic potential (ChelpG scheme) used as parameters for losartan in the molecular dynamics simulations. Atom numbers follow the labels presented in S10 Figure.

Atom		Charge	Atom		Charge
1	Cl	-0.137225	28	C	-0.049967
2	O	-0.599227	29	C	0.616585
3	N	0.004431	30	C	-0.129045
4	N	-0.546450	31	H	0.013266
5	N	-0.546855	32	H	0.039559
6	N	-0.257261	33	H	0.083553
7	N	0.316719	34	H	0.138347
8	N	-0.255322	35	H	-0.002834
9	C	0.351669	36	H	0.011179
10	C	-0.046088	37	H	-0.052673
11	C	-0.195123	38	H	-0.095311
12	C	-0.013738	39	H	-0.024542
13	C	-0.258816	40	H	0.039382
14	C	0.154960	41	H	0.117644
15	C	0.281068	42	H	0.135010
16	C	0.308582	43	H	0.036657
17	C	0.326635	44	H	0.066247
18	C	-0.179999	45	H	0.060459
19	C	-0.201446	46	H	0.061994
20	C	0.008216	47	H	0.060207
21	C	-0.042406	48	H	0.080064
22	C	-0.066354	49	H	0.381012
23	C	-0.292253	50	H	0.084041
24	C	0.170528	51	H	0.095776
25	C	-0.216070	52	H	0.101425
26	C	-0.147716	53	H	0.233138
27	C	-0.021634			
