# Supplementary material for: Insights into molecular mechanisms of drug metabolism dysfunction of human CYP2C9*30
Source: PLoS One. 2018 May 10;13(5):e0197249. doi: 10.1371/journal.pone.0197249 (PMC5944999; doi:10.1371/journal.pone.0197249)
Supplement: S3 Table — (PDF) [file pone.0197249.s013.pdf]

Table S3. Atomic parameters (bond distance, R, angle, A, and dihedral, D) for losartan obtained with quantum mechanical calculations (DFT/B3LYP/6-31G(d)). Atom labels refer to the S10 Figure numbering scheme

Atoms involved	Bond distance (Å)	Atoms involved	Angle (°)	Atoms involved	Dihedral (°)
R(1,16)	1.7436	A(17,2,49)	106.5848	D(49,2,17,13)	-59.8158
R(2,17)	1.4365	A(9,3,11)	126.6188	D(49,2,17,39)	62.2259
R(2,49)	0.9711	A(9,3,13)	107.2510	D(49,2,17,40)	177.5585
R(3,9)	1.3743	A(11,3,13)	126.1283	D(11,3,9,4)	-179.6672
R(3,11)	1.4696	A(9,4,16)	105.0308	D(11,3,9,10)	0.5727
R(3,13)	1.3962	A(7,5,29)	101.2185	D(13,3,9,4)	-0.1395
R(4,9)	1.3251	A(8,6,29)	107.0086	D(13,3,9,10)	-179.8996
R(4,16)	1.3557	A(5,7,8)	114.9670	D(9,3,11,14)	-68.1745
R(5,7)	1.3258	A(5,7,53)	122.7288	D(9,3,11,33)	54.4666
R(5,29)	1.3388	A(8,7,53)	122.2954	D(9,3,11,34)	169.6343
R(6,8)	1.3077	A(6,8,7)	105.3531	D(13,3,11,14)	112.3839
R(6,29)	1.3662	A(3,9,4)	111.4136	D(13,3,11,33)	-124.9750
R(7,8)	1.3287	A(3,9,10)	123.8703	D(13,3,11,34)	-9.8073
R(7,53)	1.0120	A(4,9,10)	124.7157	D(9,3,13,16)	0.3144
R(9,10)	1.5015	A(9,10,12)	113.2182	D(9,3,13,17)	178.0425
R(10,12)	1.5341	A(9,10,31)	109.4513	D(11,3,13,16)	179.8451
R(10,31)	1.1008	A(9,10,32)	109.4574	D(11,3,13,17)	-2.4268
R(10,32)	1.0987	A(12,10,31)	109.5360	D(16,4,9,3)	-0.1040
R(11,14)	1.5180	A(12,10,32)	109.5044	D(16,4,9,10)	179.6536
R(11,33)	1.0954	A(31,10,32)	105.3756	D(9,4,16,1)	-179.9632
R(11,34)	1.0924	A(3,11,14)	114.0051	D(9,4,16,13)	0.3228
IR(12,15)	1.5338	A(3,11,33)	108.3093	D(29,5,7,8)	-0.0424
R(12,35)	1.0974	A(3,11,34)	106.8099	D(29,5,7,53)	-178.9886
R(12,36)	1.0964	A(14,11,33)	109.8756	D(7,5,29,6)	-0.2134
R(13,16)	1.3762	A(14,11,34)	110.3787	D(7,5,29,25)	-178.02
R(13,17)	1.4893	A(33,11,34)	107.1957	D(29,6,8,7)	-0.3907
R(14,18)	1.3979	A(10,12,15)	112.2794	D(8,6,29,5)	0.3967
R(14,19)	1.4002	A(10,12,35)	109.4260	D(8,6,29,25)	178.092
R(15,23)	1.5322	A(10,12,36)	109.0093	D(5,7,8,6)	0.2841
R(15,37)	1.1000	A(15,12,35)	110.1460	D(53,7,8,6)	179.2355
R(15,38)	1.0990	A(15,12,36)	110.0026	D(3,9,10,12)	-172.7650
R(17,39)	1.0972	A(35,12,36)	105.7625	D(3,9,10,31)	-50.2587
R(17,40)	1.0948	A(3,13,16)	103.5901	D(3,9,10,32)	64.7649
R(18,21)	1.3937	A(3,13,17)	125.5054	D(4,9,10,12)	7.5067
R(18,41)	1.0877	A(16,13,17)	130.8574	D(4,9,10,31)	130.0130
R(19,22)	1.3930	A(11,14,18)	120.3471	D(4,9,10,32)	-114.9634
R(19,42)	1.0865	A(11,14,19)	120.8376	D(9,10,12,15)	-177.0502
R(20,21)	1.3996	A(18,14,19)	118.8150	D(9,10,12,35)	60.3235
R(20,22)	1.4043	A(12,15,23)	112.7570	D(9,10,12,36)	-54.9007
R(20,24)	1.4953	A(12,15,37)	109.4916	D(31,10,12,15)	60.4907
R(21,43)	1.0842	A(12,15,38)	109.4461	D(31,10,12,35)	-62.1357
R(22,44)	1.0869	A(23,15,37)	109.2943	D(31,10,12,36)	-177.3598
R(23,45)	1.0970	A(23,15,38)	109.5412	D(32,10,12,15)	-54.6063
R(23,46)	1.0970	A(37,15,38)	106.0914	D(32,10,12,35)	-177.2326
R(23,47)	1.0961	A(1,16,4)	121.2519	D(32,10,12,36)	67.5432
R(24,25)	1.4151	A(1,16,13)	126.0343	D(3,11,14,18)	117.0667

R(24,26)	1.4034	A(4,16,13)	112.7131	D(3,11,14,19)	-62.7276
R(25,27)	1.4052	A(2,17,13)	113.8416	D(33,11,14,18)	-4.7138
R(25,29)	1.4755	A(2,17,39)	110.9019	D(33,11,14,19)	175.4918
R(26,28)	1.3933	A(2,17,40)	104.7711	D(34,11,14,18)	-122.7250
R(26,48)	1.0863	A(13,17,39)	108.0062	D(34,11,14,19)	57.4806
R(27,30)	1.3909	A(13,17,40)	111.9494	D(10,12,15,23)	-179.3613
R(27,50)	1.0853	A(39,17,40)	107.1902	D(10,12,15,37)	-57.4522
R(28,30)	1.3947	A(14,18,21)	120.9409	D(10,12,15,38)	58.4461
R(28,51)	1.0866	A(14,18,41)	119.7307	D(35,12,15,23)	-57.1427
R(30,52)	1.0863	A(21,18,41)	119.3187	D(35,12,15,37)	64.7664
		A(14,19,22)	120.2308	D(35,12,15,38)	-179.3353
		A(14,19,42)	119.3610	D(36,12,15,23)	59.0552
		A(22,19,42)	120.4072	D(36,12,15,37)	-179.0357
		A(21,20,22)	118.4479	D(36,12,15,38)	-63.1373
		A(21,20,24)	121.8804	D(3,13,16,1)	179.9018
		A(22,20,24)	119.6316	D(3,13,16,4)	-0.4006
		A(18,21,20)	120.4634	D(17,13,16,1)	2.3472
		A(18,21,43)	119.7832	D(17,13,16,4)	-177.9552
		A(20,21,43)	119.7360	D(3,13,17,2)	-72.0254
		A(19,22,20)	121.0378	D(3,13,17,39)	164.3476
		A(19,22,44)	119.7087	D(3,13,17,40)	46.5699
		A(20,22,44)	119.2477	D(16,13,17,2)	105.0544
		A(15,23,45)	111.2530	D(16,13,17,39)	-18.5726
		A(15,23,46)	111.1270	D(16,13,17,40)	-136.3503
		A(15,23,47)	111.3778	D(11,14,18,21)	-177.752
		A(45,23,46)	107.4282	D(11,14,18,41)	1.1042
		A(45,23,47)	107.7415	D(19,14,18,21)	2.0464
		A(46,23,47)	107.7273	D(19,14,18,41)	-179.0974
		A(20,24,25)	123.8659	D(11,14,19,22)	177.8918
		A(20,24,26)	117.8734	D(11,14,19,42)	-2.4672
		A(25,24,26)	118.1954	D(18,14,19,22)	-1.9057
		A(24,25,27)	119.4972	D(18,14,19,42)	177.7353
		A(24,25,29)	123.5295	D(12,15,23,45)	59.9384
		A(27,25,29)	116.9167	D(12,15,23,46)	-59.7191
		A(24,26,28)	121.8248	D(12,15,23,47)	-179.8386
		A(24,26,48)	118.3250	D(37,15,23,45)	-62.0820
		A(28,26,48)	119.8501	D(37,15,23,46)	178.2606
		A(25,27,30)	121.2732	D(37,15,23,47)	58.1410
		A(25,27,50)	118.5993	D(38,15,23,45)	-177.9226
		A(30,27,50)	120.1273	D(38,15,23,46)	62.4200
		A(26,28,30)	119.6938	D(38,15,23,47)	-57.6995
		A(26,28,51)	119.8854	D(14,18,21,20)	-0.0579
		A(30,28,51)	120.4208	D(14,18,21,43)	178.4208
		A(5,29,6)	111.4512	D(41,18,21,20)	-178.9188
		A(5,29,25)	122.1753	D(41,18,21,43)	-0.4400
		A(6,29,25)	126.3324	D(14,19,22,20)	-0.2086
		A(27,30,28)	119.5127	D(14,19,22,44)	-179.3230
		A(27,30,52)	119.9240	D(42,19,22,20)	-179.8459
		A(28,30,52)	120.5633	D(42,19,22,44)	1.0398
				D(22,20,21,18)	-2.0433
				D(22,20,21,43)	179.4772
				D(24,20,21,18)	-179.7293

D(24,20,21,43)	1.7912
D(21,20,22,19)	2.1824
D(21,20,22,44)	-178.6992
D(24,20,22,19)	179.9219
D(24,20,22,44)	-0.9598
D(21,20,24,25)	-73.2768
D(21,20,24,26)	109.7228
D(22,20,24,25)	109.0639
D(22,20,24,26)	-67.9365
D(20,24,25,27)	-176.9694
D(20,24,25,29)	0.2056
D(26,24,25,27)	0.0220
D(26,24,25,29)	177.1970
D(20,24,26,28)	177.6305
D(20,24,26,48)	-2.5231
D(25,24,26,28)	0.4564
D(25,24,26,48)	-179.6972
D(24,25,27,30)	-0.4349
D(24,25,27,50)	179.4239
D(29,25,27,30)	-177.7940
D(29,25,27,50)	2.0648
D(24,25,29,5)	-141.7607
D(24,25,29,6)	40.7737
D(27,25,29,5)	35.4818
D(27,25,29,6)	-141.9838
D(24,26,28,30)	-0.5252
D(24,26,28,51)	179.5249
D(48,26,28,30)	179.6307
D(48,26,28,51)	-0.3192
D(25,27,30,28)	0.3729
D(25,27,30,52)	-179.5791
D(50,27,30,28)	-179.4838
D(50,27,30,52)	0.5642
D(26,28,30,27)	0.1038
D(26,28,30,52)	-179.9445
D(51,28,30,27)	-179.9466
D(51,28,30,52)	0.0051
